# Association of SARS-CoV-2 Infection With Psychological Distress, Psychotropic Prescribing, Fatigue, and Sleep Problems Among UK Primary Care Patients

**DOI:** 10.1001/jamanetworkopen.2021.34803

**Published:** 2021-11-16

**Authors:** Kathryn M. Abel, Matthew J. Carr, Darren M. Ashcroft, Trudie Chalder, Carolyn A. Chew-Graham, Holly Hope, Navneet Kapur, Sally McManus, Sarah Steeg, Roger T. Webb, Matthias Pierce

**Affiliations:** 1 Greater Manchester Mental Health Trust, Manchester, United Kingdom; 2 Centre for Women’s Mental Health, University of Manchester, Manchester, United Kingdom; 3 Division of Psychology and Mental Health, University of Manchester, Manchester, United Kingdom; 4 Manchester Academic Health Science Centre, Manchester, United Kingdom; 5 Centre for Pharmacoepidemiology and Drug Safety, University of Manchester, Manchester, United Kingdom; 6 National Institute for Health Research Greater Manchester Patient Safety Translational Research Centre, University of Manchester, Manchester, United Kingdom; 7 Department of Psychological Medicine, Institute of Psychiatry, Psychology & Neuroscience, King’s College London, London, United Kingdom; 8 School of Medicine, Keele University, Keele, United Kingdom; 9 National Centre for Social Research, London, United Kingdom; 10 School of Health Sciences, City, University of London, London, United Kingdom; 11 Faculty of Biology, Medicine and Health Sciences, University of Manchester, Manchester, United Kingdom

## Abstract

**Importance:**

Infection with SARS-CoV-2 is associated with fatigue and sleep problems long after the acute phase of COVID-19. In addition, there are concerns of SARS-CoV-2 infection causing psychiatric illness; however, evidence of a direct effect is inconclusive.

**Objective:**

To assess risk of risk of incident or repeat psychiatric illness, fatigue, or sleep problems following SARS-CoV-2 infection and to analyze changes according to demographic subgroups.

**Design, Setting, and Participants:**

This cohort study assembled matched cohorts using the Clinical Practice Research Datalink Aurum, a UK primary care registry of 11 923 499 individuals aged 16 years or older. Patients were followed-up for up to 10 months, from February 1 to December 9, 2020. Individuals with less than 2 years of historical data or less than 1 week follow-up were excluded. Individuals with positive results on a SARS-CoV-2 test without prior mental illness or with anxiety or depression, psychosis, fatigue, or sleep problems were matched with up to 4 controls based on sex, general practice, and year of birth. Controls were individuals who had negative SARS-CoV-2 test results. Data were analyzed from January to July 2021.

**Exposure:**

SARS-CoV-2 infection, determined via polymerase chain reaction testing.

**Main Outcomes and Measures:**

Cox proportional hazard models estimated the association between a positive SARS-CoV-2 test result and subsequent psychiatric morbidity (depression, anxiety, psychosis, or self-harm), sleep problems, fatigue, or psychotropic prescribing. Models adjusted for comorbidities, ethnicity, smoking, and body mass index.

**Results:**

Of 11 923 105 eligible individuals (6 011 020 [50.4%] women and 5 912 085 [49.6%] men; median [IQR] age, 44 [30-61] years), 232 780 individuals (2.0%) had positive result on a SARS-CoV-2 test. After applying selection criteria, 86 922 individuals were in the matched cohort without prior mental illness, 19 020 individuals had prior anxiety or depression, 1036 individuals had psychosis, 4152 individuals had fatigue, and 4539 individuals had sleep problems. After adjusting for observed confounders, there was an association between positive SARS-CoV-2 test results and psychiatric morbidity (adjusted hazard ratio [aHR], 1.83; 95% CI, 1.66-2.02), fatigue (aHR, 5.98; 95% CI, 5.33-6.71), and sleep problems (aHR, 3.16; 95% CI, 2.64-3.78). However, there was a similar risk of incident psychiatric morbidity for those with a negative SARS-CoV-2 test results (aHR, 1.71; 95% CI, 1.65-1.77) and a larger increase associated with influenza (aHR, 2.98; 95% CI, 1.55-5.75).

**Conclusions and Relevance:**

In this cohort study of individuals registered at an English primary care practice during the pandemic, there was consistent evidence that SARS-CoV-2 infection was associated with increased risk of fatigue and sleep problems. However, the results from the negative control analysis suggest that unobserved confounding may be responsible for at least some of the positive association between COVID-19 and psychiatric morbidity.

## Key Points

**Question:**

Is SARS-CoV-2 infection associated with risk of subsequent psychiatric morbidity, sleep problems, or fatigue?

**Findings:**

In this cohort study of the health care records of 11 923 105 patients, including 226 521 patients with SARS-CoV-2 infection, while infection was associated with increased risk of sleep problems and fatigue, associations with subsequent psychiatric morbidity were mixed.

**Meaning:**

These findings suggest that psychiatric morbidity associated with SARS-CoV-2 infection may be overstated in analyses of health care records that do not sufficiently control for confounding.

## Teaser

This cohort study assesses the association of SARS-CoV-2 infection with risk of incident or repeat psychiatric illness, fatigue, or sleep problems among individuals in the UK.

## Introduction

Many people infected with SARS-CoV-2 experience symptoms beyond the acute phase of COVID-19, particularly fatigue, brain fog, and sleep problems.^1,2^ Studies have also reported worsening mental health and an increased risk of psychiatric illness after COVID-19,^3,4,5,6,7,8^ and mechanisms linking the immune system, inflammation, and the brain have been proposed.^9,10^ Notably, not all studies have found an association of COVID-19 with anxiety or depression.^11^

To date, 37% of the US population has been infected with SARS-CoV-2 and only approximately 1 in 4 individuals present for testing.^12,13^ Observational studies investigating the outcomes associated with SARS-CoV-2 infection may be confounded by several sources affecting the likelihood that somebody is infected (eg, their occupation), the likelihood they present to services (eg, comorbidities), or the likelihood they seek a test (eg, health anxiety). When unobserved confounding is suspected, the extent that confounding bias is evident can be examined using a negative control.^14^ In a negative exposure control, a variable with no conceivable direct effect on the outcome, but with a similar confounding structure, is substituted for the exposure under investigation. If the result from the negative control is similar to that observed using the primary exposure, then unobserved confounding is implicated.

We examined the association between SARS-CoV-2 infection and psychiatric morbidity (ie, anxiety, depression, self-harm, psychosis, and prescription of psychotropic medication), sleep problems, and fatigue using UK primary care data. In the United Kingdom, most of health care takes place in primary care. Therefore, reexamining associations within this population might provide further detail as to the outcomes associated with COVID-19 in the population. To our knowledge, this is the first study to use a negative test result as a negative exposure control to detect unobserved confounding.

We consider both incident events as well as outcomes in people with preexisting mental illness, fatigue, or sleep problems. Our primary hypothesis was that SARS-CoV-2 infection would be associated with increased likelihood of new or repeat presentation of psychiatric morbidity, sleep problems, or fatigue independently of confounders. We also hypothesized that the increase in risk would be greatest for women and those living in more socioeconomically deprived areas. We investigated the specificity of infection with SARS-CoV-2 and psychological outcomes by repeating our analysis in individuals with an influenza diagnosis.

## Methods

For this cohort study, data access was granted following approval from the Independent Scientific Advisory Committee for Medicines and Healthcare Products Regulatory Agency Database Research.^15^ All patient data were deidentified; thus, the requirement for patient consent was waived by CRPD. Individual patients can opt out of sharing their records with the Clinical Practice Research Datalink (CPRD) Aurum. This study is prepared in line with the Reporting of Studies Conducted Using Observational Routinely-Collected Data (RECORD) statement. The protocol was preregistered before any outcomes were analyzed and is available elsewhere.^16^

### Data Source

Cohorts were assembled from the CPRD Aurum data set, a large UK primary care registry covering 19 million patients.^17^ It contains information on clinical events recorded by health care professionals, including diagnosis, symptoms, and therapies.

### Eligible Cohort

Eligible individuals were those aged 16 years or older during 2020 and registered at a CPRD Aurum participating clinical practice from February 1 to December 8, 2020. Eligible follow-up began on the latest date of February 1, 2020, or their clinical practice registration, and ended at the earliest date of their death, the date they transferred out of a clinical practice, or the end of data collection (December 9, 2020). A total of 394 individuals were excluded if they had an indeterminate sex recorded. This resulted in 11 923 105 individuals for matching.

### Exposure

Exposure to SARS-CoV-2 was defined as a positive result on a polymerase chain reaction (PCR) test using codes developed by the UK Medicines and Healthcare Products Regulatory Agency.^18^ In the United Kingdom, while most testing took place in the community, in primary care, individuals presenting with symptoms consistent with COVID-19 underwent PCR testing. In addition, since July 20, 2020, primary care physicians were notified of all PCR test results, regardless of outcome.^19^ In total, 232 780 of 11 923 105 eligible individuals (2.0%) had a record of a positive SARS-CoV-2 test result during the observation period.

### Outcome

The primary outcomes were a diagnosis with or symptoms relating to depression, anxiety disorders, self-harm, affective or nonaffective psychosis, sleep problems, and fatigue or fatigue-like syndromes (eg, postviral fatigue syndrome). There were few cases of posttraumatic stress disorder (PTSD) so, in a departure from our protocol, these were combined with anxiety disorders. Secondary outcomes were prescriptions for psychotic medications (ie, antidepressants, anxiolytics, antipsychotics, mood stabilizers, benzodiazepines, and nonbenzodiazepines hypnotics). Outcome codes were identified in a prior analysis and are published elsewhere.^20^ Two senior clinical academics (C.A.C.-G. and N.K.) reviewed the clinical code list, and a senior academic pharmacist (D.M.A.) reviewed the medication code list.

### Covariates

Covariates were identified from 10 years of patient data recorded before the start of each individual’s follow-up. Comorbidities were identified using individual diseases used for the Charlson comorbidity index^21^: cancer, cerebrovascular disease, chronic pulmonary disease, congestive heart failure, connective tissue disease, dementia, diabetes, HIV/AIDS, hemiplegia, myocardial infarction, liver disease, renal disease, peripheral vascular disease, posterior vitreous detachment, and peptic ulcer disease. Additional data were extracted on sex, year of birth, and the practice’s Index of Multiple Deprivation score, an area-level ranking of socioeconomic deprivation divided into quintiles according to the national distribution. In the UK general practices, ethnicity is self-reported during registration or at clinical consultations. There are 296 different ethnicity codes in the CPRD, and these were categorized by the analysis team (M.P. and M.C.) as White, Asian, Black, Mixed and other (eg, Arab, Cook Island Māori, and Latin American) (eTable 1 in the Supplement).

### Matched Cohorts

Five cohorts were constructed by matching individuals with a positive SARS-CoV-2 test result during follow-up with unexposed individuals (eFigure 1 in the Supplement). An individual’s earliest date of positive test result was defined as the index date. The first cohort (hereafter *incident cohort*) excluded individuals with recorded histories of psychiatric morbidity, fatigue, sleep problems, or psychotropic medications in the 5 years prior to their index date. The remaining 4 prevalent cohorts comprised individuals with common mental illness, psychosis, sleep problems, or fatigue in the last 6 months. Individuals in the common mental illness and psychosis cohorts must also have had a prescription for antidepressants, anxiolytics or antipsychotics, or mood stabilizers in the last 6 months. Individuals were not excluded from a prevalent cohort based on having a history of another mental illness, fatigue, or sleep problem. The prevalent cohorts were not mutually exclusive but were mutually exclusive with the incident cohort.

We excluded individuals with less than 1 week of eligible follow-up from their index date or less than 2 years registration prior to their index date to ensure adequate capture for prior mental illnesses. Patients were ineligible for being in the unexposed group if they had a record indicating suspected or confirmed COVID-19 in their history or in the week following the index date. Unexposed individuals may have had a positive test result at a later time, thus unexposed individuals could enter the cohort as an exposed individual at a later time. After these criteria were applied, up to 4 unexposed individuals were selected for each exposed individual, regardless of whether or not they had been selected previously (ie, matching with replacement), matched on sex, clinical practice, and year of birth. The psychosis cohort (in which there were fewer available unexposed individuals) was matched solely on sex and practice.

### Additional Cohorts

Two further cohorts were constructed. The first substituted individuals with negative SARS-CoV-2 test results for those with a positive test result (560 495 negative test cases matched with 2 232 733 comparators) and proceeded with the same analysis. The second repeated the analysis using those recorded by their general practitioner as having influenza or influenza-like symptoms and with a negative SARS-CoV-2 test result within 2 weeks. For the negative test cohort, we expected that there would be little (or no) association between receiving a negative test result (vs comparators) and any of the outcomes considered. Any divergence from a null finding would indicate potential bias from unobserved confounders. For the influenza cohort, we expected to see an increase in fatigue,^22^ but to a lesser extent than that seen for individuals with SARS-CoV-2 infection.^3^

### Statistical Analysis

The index date for the exposed individuals became the start of follow-up for all in a matched set. Censoring occurred at death, when the practice ceased collecting data, or when the individual transferred to another practice. Hazard ratios (HRs) were estimated using Cox proportional hazard models, stratified on each matched set and with weights representing the number of times an individual was in a cohort. The proportional hazard assumption was investigated by graphically examining the cumulative hazard function and the scaled Schoenfeld residuals (eFigure 2 in the Supplement).

Adjusted models included variables for ethnicity, comorbidities (as binary indicators), smoking status, body mass index (BMI, calculated as weight in kilograms divided by height in meters squared; linear and squared term) and a variable indicating how long the individual had been at a clinical practice. Effect modification was explored by including a multiplicative interaction between the exposure and (individually) the covariates: age (categorized as 16-24, 25-34, 35-49, 50-59, 60-69, 70-79, and ≥80 years), sex, Index of Multiple Deprivation quintile of the general practice, and follow-up time elapsed since index date (<1 month, 1 to <3 months, 3 to <6 months, or 6-10 months). The model using follow-up time was used to loosen the assumption of nonproportional hazards over the whole of follow-up.

There were missing data for ethnicity (2 327 219 individuals [19.5%]), smoking (881 268 individuals [7.4%]), and BMI (1 757 001 individuals [14.7%]). To retain individuals, 10 imputed data sets were estimated after matching, using chained equations. Multiple imputation models included all covariates, a variable indicating the size of the general practice, and the patient’s height or weight (if recorded). Adjusted Cox models were fitted to each imputed data set, and estimates were combined using Rubin rules.

We conducted 3 preplanned sensitivity analyses. For the first, we investigated the introduction of widespread testing by fitting an interaction between period (before vs after September 1, 2020) and the exposure. For the second sensitivity analysis, a propensity score was calculated and used as a covariate in the Cox model. For calculation of the propensity score, missing data items were given separate categories, and interactions between variables were included. The third sensitivity analysis repeated the analysis of the incident cohort but including only diagnosis codes in the outcome definition.

A post hoc sensitivity analysis was conducted after review to test robustness of findings to whether exposed and unexposed individuals were engaged with health care services. Thus, the incident matched cohort was constructed, again restricting to those who had routine clinical data (ie, BMI, ethnicity, or smoking status) recorded in the last 2 years, 1 year, and 6 months.

Stata statistical software version 16 (StataCorp) was used for all analyses and graphs were produced using R statistical software version 4.0.3 (R Project for Statistical Computing). *P* values were 2-sided, but we did not use a binary threshold to denote significance. Data were analyzed from January to July 2021.

## Results

Of 11 923 105 individuals (6 011 020 [50.4%] women and 5 912 085 [49.6%] men; median [IQR] age, 44 [30-61] years) in the eligible cohort, 232 780 (2.0%) were recorded as having a positive PCR test result for SARS-CoV-2 during their follow-up. Most positive test results occurred between April and May 2020 and October and November 2020 (eFigure 3 in the Supplement). Individuals with a positive test result were more likely than those without to be women (130 775 [56.2%] women vs 5 880 245 [50.3%]) (Table 1), in the youngest or oldest age groups (45 456 individuals [19.5%] vs 1 665 762 [14.3%] aged 16-24 years; 16 083 individuals (6.9%) vs 683 993 individuals (5.9%) aged ≥80 years), and have higher BMI (median [IQR], 26.4 [22.9-30.8] vs 25.8 [22.6-29.7]) and more recorded comorbidities (51 500 individuals [22.1%] vs 2 303 458 individuals [19.7%] with ≥1 comorbid conditions). Individuals with positive SARS-CoV-2 test results were also more likely to have a clinical record in the preceding 5 years signifying psychiatric morbidity, fatigue, sleep problems, or a psychotropic medication prescription (Table 1).

**Table 1.** Characteristics of Available Unmatched Cohort According to SARS-CoV-2 Test Results

Characteristic	Positive SARS-CoV-2 test results, No. (%)	
	Yes (n = 232 780)	No (n = 11 690 325)
Sex		
Women	130 775 (56.2)	5 880 245 (50.3)
Men	102 005 (43.8)	5 810 080 (49.7)
Age group, y		
16-24	45 456 (19.5)	1 665 762 (14.3)
25-34	41 336 (17.8)	2 208 954 (18.9)
35-49	56 983 (24.5)	2 881 606 (24.7)
50-59	39 119 (16.8)	1 827 489 (15.6)
60-69	21 359 (9.2)	1 362 308 (11.7)
70-79	12 444 (5.4)	1 060 213 (9.1)
≥80	16 083 (6.9)	683 993 (5.9)
Race and ethnicity^a^		
White	152 885 (79.5)	7 545 915 (80.3)
Asian	25 988 (13.5)	996 746 (10.6)
Black	7540 (3.9)	499 926 (5.3)
Mixed	3325 (1.7)	185 221 (2.0)
Other	2646 (1.4)	175 694 (1.9)
Missing	40 396^b^	2 286 823^b^
BMI category		
<18.5	7964 (3.9)	386 194 (3.9)
≥18.5 to <25	73 790 (36.1)	3 975 991 (39.9)
≥25 to <30	64 222 (31.4)	3 217 104 (32.3)
≥30 to <40	49 321 (24.1)	2 058 755 (20.7)
≥40	9106 (4.5)	323 657 (3.3)
Missing	28 377^b^	1 728 624^b^
Time at clinical practice, median (IQR), y	9.6 (2.3-21.0)	9.4 (2.7-21.4)
Comorbidities, No.^c^		
0	181 280 (77.9)	9 386 867 (80.3)
1	26 143 (11.2)	1 332 044 (11.4)
2-4	23 262 (10.0)	922 004 (7.9)
≥5	2095 (0.9)	49 410 (0.4)
Psychiatric morbidity in the last 5 y		
Depression	38 727 (16.6)	1 601 431 (13.7)
Anxiety	31 072 (13.4)	1 271 468 (10.9)
Psychosis	2941 (1.3)	136 001 (1.2)
Eating disorder	1344 (0.6)	47 018 (0.4)
Personality disorder	726 (0.3)	42 531 (0.4)
Self-harm	2975 (1.3)	137 826 (1.2)
Fatigue	18 396 (7.9)	665 652 (5.7)
Sleep disorder	16 759 (7.2)	669 528 (5.7)
Medication in the last 5 y		
Antidepressants	65 364 (28.1)	2 681 383 (22.9)
Benzodiazepines	20 571 (8.8)	826 794 (7.1)
Nonbenzodiazepine hypnotics	12 596 (5.4)	542 565 (4.6)
Antipsychotics	5228 (2.3)	209 463 (1.8)
Mood stabilizers	15 306 (6.6)	586 523 (5.0)

^a^ Race and ethnicity categorized from 296 clinical codes. Other race or ethnicity includes Arab, Cook Island Māori, and Latin American. A full list is provided in eTable 1 in the Supplement.

^b^ Missing data excluded from percentages.

^c^ List of comorbidities from the Charlson comorbidity index.

### Outcomes in Individuals Without Prior Mental Illness, Fatigue, or Sleep Problems (Incident Cohort)

The median (IQR) follow-up of the incident cohort was 6.3 (4.0-9.3) weeks. After matching on age, sex, and registered practice, and adjusting for ethnicity, smoking status, BMI, and comorbidities, having a positive result on a SARS-CoV-2 test was associated with an increase in risk of any psychiatric morbidity (adjusted HR [aHR], 1.83; 95% CI, 1.66-2.02) and of being prescribed psychotropic medication (aHR, 2.24; 95% CI, 2.09-2.40) (Table 2). The absolute risks were low: an estimated 1.4% of patients with a positive SARS-CoV-2 test result presented with psychiatric morbidity at 6 months, compared with 0.9% of individuals with a negative test result (eTable 2 in the Supplement).

**Table 2.** Comparison of Incident Outcomes Between Patients With a Positive SARS-CoV-2 Test Result and Controls, Matched on Year of Birth, Sex, and General Practice

Outcome	Group	Events	Rate, per 1000 person-years	Hazard ratio (95% CI)	
				Unadjusted	Adjusted^a^
Any psychiatric morbidity	SARS-CoV-2	447	30.24	1.80 (1.63-1.98)	1.83 (1.66-2.02)
	Unexposed	1045	17.39	1 [Reference]	1 [Reference]
Depression	SARS-CoV-2	230	15.52	1.71 (1.50-1.95)	1.74 (1.51-2.00)
	Unexposed	571	9.49	1 [Reference]	1 [Reference]
Anxiety	SARS-CoV-2	298	20.12	1.85 (1.65-2.08)	1.93 (1.71-2.18)
	Unexposed	671	11.15	1 [Reference]	1 [Reference]
Psychosis	SARS-CoV-2	21	1.41	2.34 (1.48-3.70)	1.84 (0.93-3.64)
	Unexposed	36	0.60	1 [Reference]	1 [Reference]
Self-harm	SARS-CoV-2	13	0.87	2.09 (1.20-3.64)	2.21 (1.11-4.39)
	Unexposed	25	0.41	1 [Reference]	1 [Reference]
Sleep disorders	SARS-CoV-2	190	12.82	3.31 (2.82-3.89)	3.16 (2.64-3.78)
	Unexposed	236	3.92	1 [Reference]	1 [Reference]
Fatigue	SARS-CoV-2	579	39.29	6.10 (5.47-6.80)	5.98 (5.33-6.71)
	Unexposed	414	6.88	1 [Reference]	1 [Reference]
Any psychotropic medication	SARS-CoV-2	1055	72.05	2.41 (2.26-2.57)	2.24 (2.09-2.40)
	Unexposed	1848	30.82	1 [Reference]	1 [Reference]
Antidepressants	SARS-CoV-2	583	39.54	1.79 (1.65-1.95)	1.72 (1.57-1.88)
	Unexposed	1358	22.62	1 [Reference]	1 [Reference]
Benzodiazepines	SARS-CoV-2	266	17.95	3.84 (3.33-4.42)	3.50 (2.95-4.15)
	Unexposed	305	5.07	1 [Reference]	1 [Reference]
Nonbenzodiazepine hypnotics	SARS-CoV-2	173	11.67	4.95 (4.10-5.97)	4.90 (4.00-5.99)
	Unexposed	148	2.46	1 [Reference]	1 [Reference]
Antipsychotics	SARS-CoV-2	83	5.59	6.73 (4.99-9.08)	7.61 (5.00-11.60)
	Unexposed	63	1.05	1 [Reference]	1 [Reference]
Mood stabilizers	SARS-CoV-2	105	7.08	3.80 (3.04-4.75)	3.55 (2.74-4.61)
	Unexposed	115	1.91	1 [Reference]	1 [Reference]

^a^ Adjusted for race and ethnicity, smoking, body mass index, and comorbidities and after multiple imputation.

For almost all outcomes considered, positive SARS-CoV-2 test results were associated with increased risk. The largest increases were for receipt of antipsychotics (aHR, 7.61; 95% CI, 5.00-11.60), fatigue (aHR, 5.98; 95% CI, 5.33-6.71), receipt of nonbenzodiazepine hypnotics (aHR, 4.90; 95% CI, 4.00-5.99), receipt of mood stabilizers (a HR, 3.55; 95% CI, 2.74-4.61), and sleep problems (aHR, 3.16; 95% CI, 2.64-3.78).

There was effect modification by age, such that the association between SARS-CoV-2 infection and psychiatric morbidity was greater for older age groups (eg, ≥80 years: aHR, 4.17; 95% CI, 2.67-6.53 vs 16-24 years: aHR, 1.28; 95% CI, 1.06-1.55). For fatigue and sleep disorder, the association was greatest for those aged 60 to 69 years and remained elevated for all groups (Table 3). For all outcomes, women with positive SARS-CoV-2 test results had a higher incidence than men; however, the relative increase associated with a positive test result was larger for men. There was no association of effect modification by deprivation quintile. For sleep or psychiatric morbidity, the association was similar over follow-up.

**Table 3.** Association Between SARS-CoV-2 and Psychiatric Morbidity, Fatigue, and Sleep Disorders According to Subgroups

Group	Person-years^a^		Outcome												
			Any psychiatric morbidity				Fatigue				Sleep				
	SARS-CoV-2	Unexposed	SARS-CoV-2, rate^a^	Unexposed, rate^a^	aHR (95% CI)^b^	*P* value^c^	SARS-CoV-2, rate^a^	Unexposed, rate^a^	aHR (95% CI)^b^	*P* value^c^	SARS-CoV-2, rate^a^	Unexposed, rate^a^	aHR (95% CI)^b^	*P* value^c^
Sex														
Women	7.0	28.5	37.11	24.06	1.63 (1.43-1.85)	.003	43.01	8.68	5.25 (4.54-6.06)	.003	8.31	3.50	2.34 (1.78-3.06)	.003
Men	7.8	31.5	24.14	11.53	2.20 (1.88-2.57)		27.93	3.67	7.46 (6.19-9.01)		12.45	3.07	4.06 (3.18-5.18)	
Age-group, y														
16-24	2.6	10.9	41.69	34.49	1.28 (1.06-1.55)	<.001	14.46	5.43	2.92 (2.14-4.00)	<.001	4.32	2.55	2.08 (1.25-3.46)	.002
25-34	2.4	9.7	31.01	21.29	1.59 (1.26-2.02)		20.37	5.17	4.24 (3.14-5.73)		5.32	2.51	2.35 (1.48-3.75)	
35-49	3.8	15.0	27.92	15.20	2.00 (1.64-2.45)		39.67	5.79	6.49 (5.26-8.03)		9.72	3.05	2.99 (2.16-4.13)	
50-59	2.9	11.4	25.18	11.80	2.24 (1.75-2.88)		42.38	4.80	8.74 (6.78-11.26)		12.94	2.69	4.45 (3.00-6.59)	
60-69	1.6	6.3	22.91	7.91	3.09 (2.16-4.43)		38.94	3.95	9.15 (6.39-13.10)		11.96	1.96	7.97 (4.56-13.94)	
70-79	0.8	3.4	21.78	5.90	3.66 (2.00-6.68)		37.80	4.74	7.23 (4.51-11.60)		9.12	2.97	3.40 (1.41-8.17)	
≥80	0.7	3.2	46.06	11.97	4.17 (2.67-6.53)		32.17	9.76	5.07 (3.02-8.50)		11.50	5.88	1.38 (0.60-3.18)	
IMD quintile														
Highest	1.8	7.2	21.93	15.21	1.54 (1.13-2.10)	.30	36.38	5.44	5.86 (4.34-7.91)	.06	5.78	2.16	3.11 (1.77-5.46)	.93
Second	2.3	9.4	33.39	18.14	1.87 (1.47-2.38)		43.24	4.51	8.66 (6.53-11.48)		7.21	2.45	3.53 (2.13-5.84)	
Third	2.2	9.1	32.91	16.96	2.01 (1.58-2.56)		29.83	4.49	6.67 (4.89-9.12)		9.65	3.09	3.43 (2.15-5.47)	
Fourth	2.9	11.8	28.56	19.39	1.57 (1.25-1.96)		28.98	6.80	5.04 (3.95-6.43)		11.84	3.57	3.24 (2.24-4.70)	
Lowest	3.6	14.9	33.02	16.61	2.06 (1.70-2.51)		32.45	5.82	5.18 (4.13-6.51)		8.82	3.31	2.76 (1.96-3.90)	
Follow-up time, mo														
<1	6.1	24.5	22.02	15.78	1.57 (1.34-1.85)	.24	47.54	5.56	8.12 (6.71-9.82)	<.001	8.58	2.66	3.27 (2.33-4.61)	.12
1-3	5.3	21.5	31.58	17.69	1.97 (1.70-2.29)		24.66	5.64	4.47 (3.54-5.63)		8.75	3.57	2.27 (1.59-3.24)	
3-6	2.4	10.0	24.85	13.17	2.15 (1.68-2.75)		25.76	6.33	4.66 (3.35-6.49)		13.87	3.31	3.96 (2.49-6.31)	
≥6	0.9	4.0	14.75	8.56	1.91 (1.20-3.03)		17.49	5.61	3.03 (1.77-5.20)		12.13	2.42	5.33 (2.40-11.82)	

Abbreviation: aHR, adjusted hazard ratio.

^a^ Person-years in thousands. Rate per 1000 person-years.

^b^ Adjusted models included the covariates: ethnicity, comorbidities, smoking status, and body mass index.

^c^ *P* value testing for equivalence of aHRs.

### Outcomes in Individuals With Prior History of Common Mental Illness, Psychosis, Fatigue, and Sleep Problems

**Figure 1.**
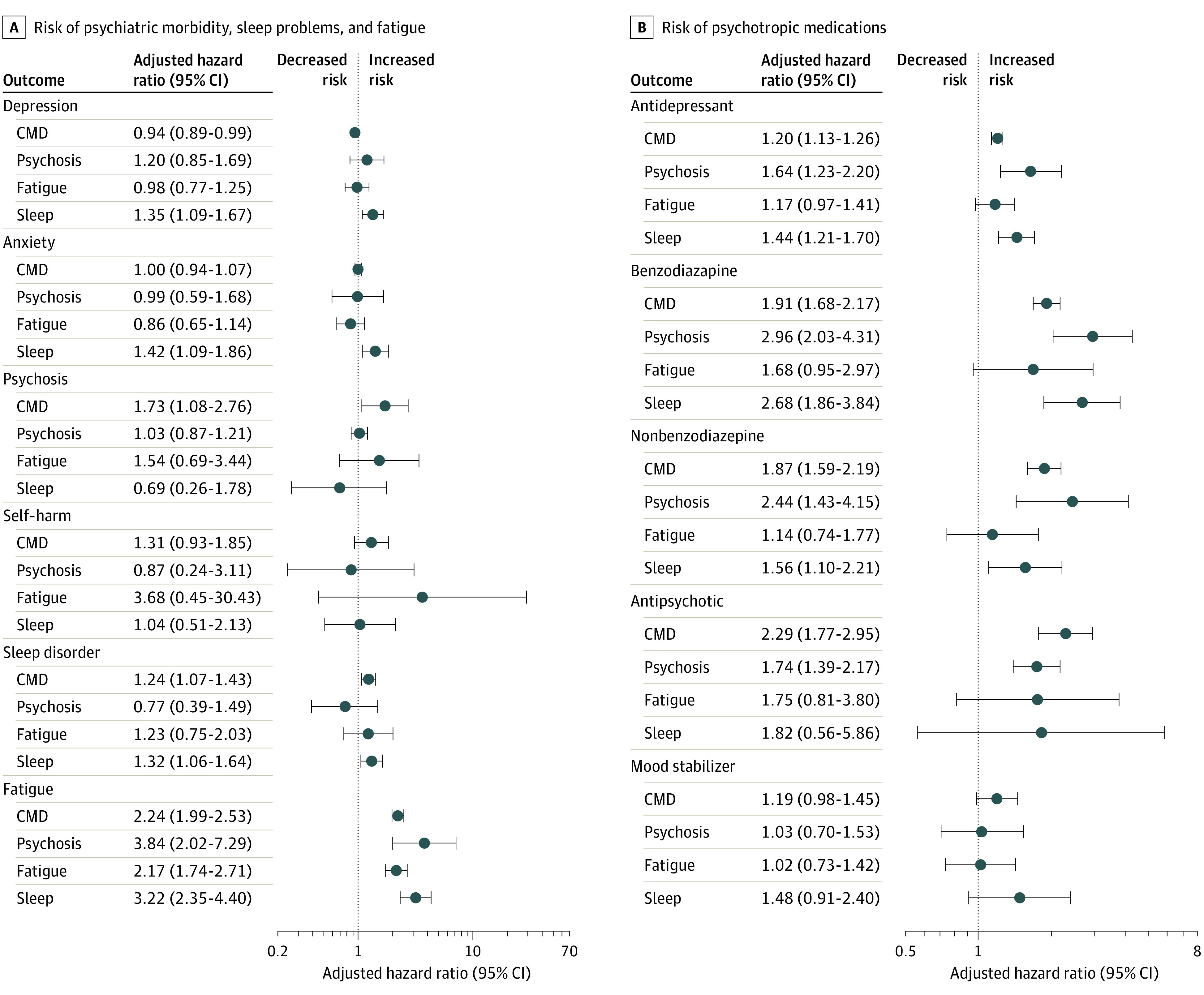
Association of Having Positive SARS-CoV-2 Test Results With Psychiatric Morbidity, Sleep Problems, Fatigue, and Psychotropic Medications CMD indicates common mental disorders. Estimates are provided in eTable 3 in the Supplement.

For those with preexisting common mental illness (ie, depression or anxiety disorders), there was no association of a positive SARS-CoV-2 test result with increased risk of subsequent depression or anxiety events (Figure 1; eTable 3 in the Supplement). However, there was an increase in the risk of new prescriptions for antidepressants (aHR, 1.20; 95% CI, 1.13-1.26) and a larger increase for new prescriptions for benzodiazepines (aHR, 1.91; 95% CI, 1.68-2.17), and a positive SARS-CoV-2 test result was associated with more than 2-fold higher risk of subsequent fatigue (aHR, 2.24; 95% CI, 1.99-2.53).

There was no evidence of an increased risk of depression or anxiety associated with SARS-CoV-2 infection for those with preexisting psychosis or fatigue. There was an increased risk of depression and anxiety disorders associated with having a positive SARS-CoV-2 test result for those with preexisting sleep problems (depression: aHR, 1.35; 95% CI, 1.09-1.67; anxiety disorders: aHR, 1.42; 95% CI, 1.09-1.86) and an increased risk of subsequent fatigue associated with having a positive SARS-CoV-2 test result for all matched cohorts.

### Negative Test Results and Influenza Cohorts

**Figure 2.**
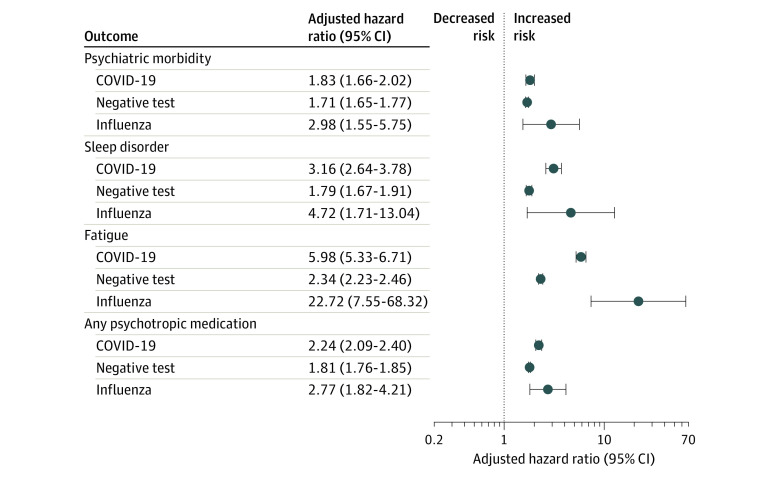
Adjusted Hazard Ratios From Incident COVID-19, Negative Exposure, and Influenza Cohorts Psychotropic medications that were prescribed 6 months prior to index date were excluded. Estimates are provided in eTable 4 in the Supplement.

Among individuals with a negative SARS-CoV-2 test result, there was an increased risk of psychiatric morbidity compared with matched controls in the general population (Figure 2). There was a similar association to the main analysis between having a negative test result and the risk of all subcategories of psychiatric morbidity, fatigue, and sleep problems (eTable 4 in the Supplement). There was an association between having an influenza-like illness and either incident psychiatric morbidity, sleep, fatigue, or psychotropic prescribing. Prior to matching, individuals with a negative SARS-CoV-2 test result and those with influenza over follow-up had a higher rate of prior mental illness than those in the group with positive SARS-CoV-2 test results (eTable 5 in the Supplement).

### Sensitivity Analyses

There were some significant interactions between period of testing and the positive test result. For individuals who had positive test results prior to September 1, 2020, there was a stronger association between having a positive SARS-CoV-2 test result and psychiatric morbidity or psychotropic medication than those who had positive results on or after September 1, 2020 (eTable 6 in the Supplement). The estimates, after adjusting for a propensity score, were of a similar magnitude to that seen in the main analysis (eTable 7 in the Supplement). The estimates for anxiety and depression were also similar after including only diagnostic codes in the outcome; however, for psychosis, there was little evidence of an increase associated with SARS-CoV-2 infection (eTable 8 in the Supplement). When the cohort was restricted to those with recent clinical contact, there were similar associations to those seen in the main analysis (eTable 9 in the Supplement).

## Discussion

In this cohort study, we found that SARS-CoV-2 infection confirmed with a positive PCR test result was associated with increased risk of incident psychiatric morbidity, sleep problems, and fatigue in the following months. However, sensitivity analyses provided doubt about whether some of these outcomes were directly associated with SARS-CoV-2 infection. Notably, using the same processes as the analysis of patients with a positive SARS-CoV-2 test result (eg, selection criteria, confounding control), individuals with a negative SARS-CoV-2 test result also experienced a substantial increase in risk of incident psychiatric morbidity. This association was of similar magnitude and with overlapping CIs to that observed in people with a positive SARS-CoV-2 test results. Having a negative test result for SARS-CoV-2, in and of itself, is unlikely to cause psychiatric morbidity through any direct mechanism; rather, this indicates there are unobserved confounders linking the likelihood of undergoing a test and the likelihood of having an incident psychiatric episode during the COVID-19 pandemic.

We believe the most likely unobserved confounding variables are occupation and health anxiety. While many individuals were placed on furlough or worked from home, many sectors of work (eg, health care workers) were required to continue as usual, raising the likelihood of exposure to SARS-CoV-2 and potentially leading to excess psychological strain.^23^ In addition, those who seek a test for COVID-19 symptoms could already be experiencing health anxieties that might indicate future mental illness. Those with a negative test result had a higher proportion of prior mental illness than those with a positive test, perhaps indicating that health anxiety might be more strongly associated with having a negative test result rather than a positive one. It has been estimated that approximately three-fourths of individuals infected with SARS-CoV-2 have not been tested,^13^ and the likelihood of seeking a test may be guided by similar behavioral factors regardless of whether the results would be positive or negative. The negative exposure control analysis showed these factors should not be ignored, and while this does not rule out a direct association of SARS-CoV-2 infection with subsequent psychiatric morbidity, it provides substantial doubt.

We included a separate investigation of individuals exposed to influenza during the same periods to examine the specificity of the association of incident psychiatric morbidity, fatigue, and sleep problems with SARS-CoV-2 infection as opposed to other respiratory diseases. For all outcomes, the increase in risk was considerably larger for individuals presenting for primary care with influenza than with SARS-CoV-2 infection. This is likely because these form a particularly selective group: overall there were fewer cases of influenza observed during the COVID-19 pandemic,^24^ and individuals who present with influenza may have been more likely to have severe infection, preexisting morbidity, and/or psychological risk factors.

Our finding contrasts with 2 recent studies using US administrative data^3,4^ that reported that individuals with positive test results for SARS-CoV-2 had approximately 2-fold higher risk of subsequent psychiatric illness compared with those with influenza infection. There are some key design differences that may explain this: the US analysis did not follow-up individuals with SARS-CoV-2 infection and the controls from the same date, nor did they adjust for geographical area. Because the pandemic has ecological effects on localized health care systems, as well as on population mental health, this could introduce substantive biases.^25^ Also, the US may have confounding that is less evident in the United Kingdom. For example, in the US, areas with high levels of socioeconomic deprivation and inequality had significantly higher incidence of COVID-19,^26,27^ these areas are also likely to have higher rates of mental illness.^28,29^

Our analysis is in agreement with a Danish registry study that did not find an association between having positive SARS-CoV-2 test results, vs negative test results, and subsequent mental illness.^11^ This analysis excluded patients hospitalized for COVID-19 (which we were unable to do); however, we note that the US study^3^ reported similar estimates for those who were hospitalized and those not.

### Limitations

There are several limitations to our study, besides unobserved confounding. First, approximately 8.7% of the population of England had positive results in antibody testing for SARS-CoV-2 by December 2020,^30^ indicating they had previously been infected, compared with 2.0% of individuals in the study population who had positive results in SARS-CoV-2 PCR testing. Therefore, the comparison group will have contained many who had a SARS-CoV-2 infection but were asymptomatic or were symptomatic and did not report it. This misclassification will likely bias results toward the null. Second, because testing was relatively scarce during the early phases of the pandemic and there was no requirement automatically to notify general practices until July 20, 2020, most patients with SARS-CoV-2 infection were identified during the United Kingdom’s second wave of infections (ie, November-December 2020); therefore, the median follow-up was relatively short (6.3 weeks). Additionally, the cohort is based on administrative records, rather than active follow-up, and therefore will have contained some people who moved away. However, the results were sensitive to participants having recent clinical contact.

## Conclusions

This cohort study of individuals with a positive SARS-CoV-2 PCR test result found an association of SARS-CoV-2 infection with fatigue and sleep problems; however there is some doubt about a direct association of SARS-CoV-2 with psychiatric outcomes. Other designs may be more appropriate to investigate the association of SARS-CoV-2 infection with mental health outcomes. For example, individuals, randomly selected, who took part in serological surveys might be more representative of the general population and, thus, less susceptible to bias.

## Notes

Supplement. **eTable 1.** Categorization of Race and Ethnicity From the Clinical Codes in CPRD **eFigure 1.** Flowchart Showing Selection in to Matched Cohorts **eFigure 2.** Cumulative Hazard and Scaled Schoenfeld Residual Plots **eFigure 3.** Histogram of Frequency of Positive SARS-CoV-2 Test Results by Date During 2020 **eTable 2.** Proportion of Individuals With Positive Test Results and Controls With Outcomes After 6 Months Within Each Matched Cohort **eTable 3.** Psychiatric Morbidity, Sleep Problems, Fatigue and Uniquely Prescribed Psychotropic Medications for Those With Preexisting Common Mental Illness, Psychosis, Fatigue, or Sleep Problems Matched on Year of Birth, Sex, and General Practice **eTable 4.** Comparison of Adjusted Hazard Ratios From Matched Positive SARS-CoV-2 Test Results, Negative SARS-CoV-2 Test Results, and Influenza Cohorts **eTable 5.** Description of Eligible Cohort According to SARS-CoV-2 Test and Influenza Status Over Follow-up **eTable 6.** Estimates for Individuals With Positive SARS-CoV-2 Test Results in the First or Second Wave **eTable 7.** Comparison of Estimates From Main Adjusted Analysis of the Incident Cohort With That Calculated Controlling for a Propensity Score **eTable 8.** Estimates From the Main Analysis for Depression, Anxiety, and Psychosis and After Including Only Diagnosis Codes in the Outcome Definition **eTable 9.** Repeating the Incident Matched Analysis for Individuals With Recent Clinical Contact, as Indicated by Recording of Routine Clinical Data

## References

[1] Office for National Statistics. The prevalence of long COVID symptoms and COVID-19 complications. Updated December 2020. Accessed October 18, 2021. https://www.ons.gov.uk/news/statementsandletters/theprevalenceoflongcovidsymptomsandcovid19complications

[2] López-León S, Wegman-Ostrosky T, Perelman C, . More than 50 long-term effects of COVID-19: a systematic review and meta-analysis. Sci Rep. 2021;11(1):16144. doi:10.1038/s41598-021-95565-8 34373540 PMC8352980

[3] Taquet M, Luciano S, Geddes JR, Harrison PJ. Bidirectional associations between COVID-19 and psychiatric disorder: retrospective cohort studies of 62 354 COVID-19 cases in the USA. Lancet Psychiatry. 2021;8(2):130-140. doi:10.1016/S2215-0366(20)30462-4 33181098 PMC7820108

[4] Taquet M, Geddes JR, Husain M, Luciano S, Harrison PJ. 6-month neurological and psychiatric outcomes in 236 379 survivors of COVID-19: a retrospective cohort study using electronic health records. Lancet Psychiatry. 2021;8(5):416-427. doi:10.1016/S2215-0366(21)00084-5 33836148 PMC8023694

[5] Niedzwiedz CL, Benzeval M, Hainey K, Leyland AH, Katikireddi SV. Psychological distress among people with probable COVID-19 infection: analysis of the UK Household Longitudinal Study. BJPsych Open. 2021;7(3):e104. doi:10.1192/bjo.2021.63 34001295 PMC8134894

[6] Daugherty SE, Guo Y, Heath K, . Risk of clinical sequelae after the acute phase of SARS-CoV-2 infection: retrospective cohort study. BMJ. 2021;373:n1098. doi:10.1136/bmj.n1098 34011492 PMC8132065

[7] Al-Aly Z, Xie Y, Bowe B. High-dimensional characterization of post-acute sequelae of COVID-19. Nature. 2021;594(7862):259-264. doi:10.1038/s41586-021-03553-9 33887749

[8] Pierce M, McManus S, Hope H, . Mental health responses to the COVID-19 pandemic: a latent class trajectory analysis using longitudinal UK data. Lancet Psychiatry. 2021;8(7):610-619. doi:10.1016/S2215-0366(21)00151-6 33965057 PMC9764381

[9] Mazza MG, De Lorenzo R, Conte C, ; COVID-19 BioB Outpatient Clinic Study group. Anxiety and depression in COVID-19 survivors: role of inflammatory and clinical predictors. Brain Behav Immun. 2020;89(July):594-600. doi:10.1016/j.bbi.2020.07.037 32738287 PMC7390748

[10] Rogers JP, Chesney E, Oliver D, . Psychiatric and neuropsychiatric presentations associated with severe coronavirus infections: a systematic review and meta-analysis with comparison to the COVID-19 pandemic. Lancet Psychiatry. 2020;7(7):611-627. doi:10.1016/S2215-0366(20)30203-0 32437679 PMC7234781

[11] Lund LC, Hallas J, Nielsen H, . Post-acute effects of SARS-CoV-2 infection in individuals not requiring hospital admission: a Danish population-based cohort study. Lancet Infect Dis. 2021;21(10):1373-1382. doi:10.1016/S1473-3099(21)00211-5 33984263 PMC8110209

[12] Smith LE, Potts HWW, Amlôt R, Fear NT, Michie S, Rubin GJ. Adherence to the test, trace, and isolate system in the UK: results from 37 nationally representative surveys. BMJ. 2021;372(n608):n608. doi:10.1136/bmj.n608 33789843 PMC8010268

[13] Centers for Disease Control and Prevention. Estimated COVID-19 burden. Updated July 2021. Accessed August 23, 2021. https://www.cdc.gov/coronavirus/2019-ncov/cases-updates/burden.html

[14] Lipsitch M, Tchetgen Tchetgen E, Cohen T. Negative controls: a tool for detecting confounding and bias in observational studies. Epidemiology. 2010;21(3):383-388. doi:10.1097/EDE.0b013e3181d61eeb 20335814 PMC3053408

[15] CPRD. Clinical contact with health services for mental illness and self-harm before, during and after the COVID-19 pandemic. Accessed October 18, 2021. https://cprd.com/protocol/clinical-contact-health-services-mental-illness-and-self-harm-during-and-after-covid-19

[16] Center for Open Science. COVID-19 infection and risk of developing a mental illness, fatigue symptoms, sleep problems or self-harming behaviour. Accessed October 21, 2021. https://osf.io/rs9d8/

[17] Wolf A, Dedman D, Campbell J, . Data resource profile: Clinical Practice Research Datalink (CPRD) Aurum. Int J Epidemiol. 2019;48(6):1740-1740g. doi:10.1093/ije/dyz034 30859197 PMC6929522

[18] Medicines & Healthcare Products Regulatory Agency. Feasibility counts for SARS-CoV-2-related codes in CPRD primary care data. Accessed October 18, 2021. https://www.cprd.com/sites/default/files/SARS-CoV-2%20counts%20June21_v1.0.pdf

[19] Coronavirus test results now visible to GPs. News release. NHS Digital. July 20, 2020. Accessed May 24, 2021. https://digital.nhs.uk/news-and-events/latest-news/coronavirus-test-results-now-visible-to-gps

[20] Steeg S. Primary care contact for mental illness and self-harm before, during and after the peak of the COVID-19 pandemic in the UK: cohort study of 13 million individuals. Accessed October 18, 2021. https://clinicalcodes.rss.mhs.man.ac.uk/medcodes/article/173/

[21] Charlson M, Szatrowski TP, Peterson J, Gold J. Validation of a combined comorbidity index. J Clin Epidemiol. 1994;47(11):1245-1251. doi:10.1016/0895-4356(94)90129-5 7722560

[22] Wessely S, Chalder T, Hirsch S, Pawlikowska T, Wallace P, Wright DJ. Postinfectious fatigue: prospective cohort study in primary care. Lancet. 1995;345(8961):1333-1338. doi:10.1016/S0140-6736(95)92537-6 7752755

[23] Serrano-Ripoll MJ, Meneses-Echavez JF, Ricci-Cabello I, . Impact of viral epidemic outbreaks on mental health of healthcare workers: a rapid systematic review and meta-analysis. J Affect Disord. 2020;277(August):347-357. doi:10.1016/j.jad.2020.08.034 32861835 PMC7443314

[24] Public Health England (PHE). PHE national influenza report: Summary of UK surveillance of influenza and other seasonal respiratory illnesses—06 August 2020—week 32 report. Accessed October 18, 2021. https://assets.publishing.service.gov.uk/government/uploads/system/uploads/attachment_data/file/907349/National_Influenza_report_06_August_2020_week_32.pdf

[25] Accorsi EK, Qiu X, Rumpler E, . How to detect and reduce potential sources of biases in studies of SARS-CoV-2 and COVID-19. Eur J Epidemiol. 2021;36(2):179-196. doi:10.1007/s10654-021-00727-7 33634345 PMC7906244

[26] Adhikari S, Pantaleo NP, Feldman JM, Ogedegbe O, Thorpe L, Troxel AB. Assessment of community-level disparities in coronavirus disease 2019 (COVID-19) infections and deaths in large US metropolitan areas. JAMA Netw Open. 2020;3(7):e2016938. doi:10.1001/jamanetworkopen.2020.16938 32721027 PMC7388025

[27] Liao TF, De Maio F. Association of social and economic inequality with coronavirus disease 2019 incidence and mortality across US counties. JAMA Netw Open. 2021;4(1):e2034578. doi:10.1001/jamanetworkopen.2020.34578 33471120 PMC7818127

[28] Ribeiro WS, Bauer A, Andrade MCR, . Income inequality and mental illness-related morbidity and resilience: a systematic review and meta-analysis. Lancet Psychiatry. 2017;4(7):554-562. doi:10.1016/S2215-0366(17)30159-1 28552501

[29] Allen J, Balfour R, Bell R, Marmot M. Social determinants of mental health. Int Rev Psychiatry. 2014;26(4):392-407. doi:10.3109/09540261.2014.928270 25137105

[30] Office for National Statistics. Coronavirus (COVID-19) Infection Survey: characteristics of people testing positive for COVID-19 in England and antibody data for the UK. Accessed October 18, 2021. https://www.ons.gov.uk/peoplepopulationandcommunity/healthandsocialcare/conditionsanddiseases/articles/coronaviruscovid19infectionsinthecommunityinengland/december2020

