# Supplementary material for: Association of SARS-CoV-2 Infection With Psychological Distress, Psychotropic Prescribing, Fatigue, and Sleep Problems Among UK Primary Care Patients
Source: JAMA Netw Open. 2021 Nov 16;4(11):e2134803. doi: 10.1001/jamanetworkopen.2021.34803 (PMC8596199; doi:10.1001/jamanetworkopen.2021.34803)
Supplement: Supplement. — eTable 1. Categorization of Race and Ethnicity From the Clinical Codes in CPRD eFigure 1. Flowchart Showing Selection in to Matched Cohorts eFigure 2. Cumulative Hazard and Scaled Schoenfeld Residual Plots eFigure 3. Histogram of Frequency of Positive SARS-CoV-2 Test Results by Date During 2020 eTable 2. Proportion of Individuals With Positive Test Results and Controls With Outcomes After 6 Months Within Each Matched Cohort eTable 3. Psychiatric Morbidity, Sleep Problems, Fatigue and Uniquely Prescribed Psychotropic Medications for Those With Preexisting Common Mental Illness, Psychosis, Fatigue, or Sleep Problems Matched on Year of Birth, Sex, and General Practice eTable 4. Comparison of Adjusted Hazard Ratios From Matched Positive SARS-CoV-2 Test Results, Negative SARS-CoV-2 Test Results, and Influenza Cohorts eTable 5. Description of Eligible Cohort According to SARS-CoV-2 Test and Influenza Status Over Follow-up eTable 6. Estimates for Individuals With Positive SARS-CoV-2 Test Results in the First or Second Wave eTable 7. Comparison of Estimates From Main Adjusted Analysis of the Incident Cohort With That Calculated Controlling for a Propensity Score eTable 8. Estimates From the Main Analysis for Depression, Anxiety, and Psychosis and After Including Only Diagnosis Codes in the Outcome Definition eTable 9. Repeating the Incident Matched Analysis for Individuals With Recent Clinical Contact, as Indicated by Recording of Routine Clinical Data [file jamanetwopen-e2134803-s001.pdf]

Supplemental Online Content

Abel KM, Carr MJ, Ashcroft DM, et al. Association of SARS-CoV-2 infection with psychological distress, psychotropic prescribing, fatigue, and sleep problems among UK primary care patients. *JAMA Netw Open*. 2021;4(11):e2134803. doi:10.1001/jamanetworkopen.2021.34803

eTable 1. Categorization of Race and Ethnicity From the Clinical Codes in CPRD

eFigure 1. Flowchart Showing Selection in to Matched Cohorts

eFigure 2. Cumulative Hazard and Scaled Schoenfeld Residual Plots

eFigure 3. Histogram of Frequency of Positive SARS-CoV-2 Test Results by Date During 2020

eTable 2. Proportion of Individuals With Positive Test Results and Controls With Outcomes After 6 Months Within Each Matched Cohort

eTable 3. Psychiatric Morbidity, Sleep Problems, Fatigue and Uniquely Prescribed Psychotropic Medications for Those With Preexisting Common Mental Illness, Psychosis, Fatigue, or Sleep Problems Matched on Year of Birth, Sex, and General Practice

eTable 4. Comparison of Adjusted Hazard Ratios From Matched Positive SARS-CoV-2 Test Results, Negative SARS-CoV-2 Test Results, and Influenza Cohorts

eTable 5. Description of Eligible Cohort According to SARS-CoV-2 Test and Influenza Status Over Follow-up

eTable 6. Estimates for Individuals With Positive SARS-CoV-2 Test Results in the First or Second Wave

eTable 7. Comparison of Estimates From Main Adjusted Analysis of the Incident Cohort With That Calculated Controlling for a Propensity Score

eTable 8. Estimates From the Main Analysis for Depression, Anxiety, and Psychosis and After Including Only Diagnosis Codes in the Outcome Definition

eTable 9. Repeating the Incident Matched Analysis for Individuals With Recent Clinical Contact, as Indicated by Recording of Routine Clinical Data

This supplemental material has been provided by the authors to give readers additional information about their work.

© 2021 Abel KM et al. *JAMA Network Open.*

eTable 1. Categorization of Race and Ethnicity From the Clinical Codes in CPRD

Clinical code description	Category
African - ethnic category 2001 census	Black
African: African, African Scottish or African British - Scotland ethnic category 2011 census	Black
African: any other African - Scotland ethnic category 2011 census	Black
Afro-Caribbean	Black
Afro-Caucasian	Black
Albanian - ethnic category 2001 census	Other
Any other group - ethnic category 2001 census	Other
Arab - ethnic category 2001 census	Other
Arabs	Other
Asian - ethnic group	Asian
Asian and Chinese - ethnic category 2001 census	Asian
Asian or Asian British: Bangladeshi - England and Wales ethnic category 2011 census	Asian
Asian or Asian British: Bangladeshi - Northern Ireland ethnic category 2011 census	Asian
Asian or Asian British: Chinese - England and Wales ethnic category 2011 census	Asian
Asian or Asian British: Chinese - Northern Ireland ethnic category 2011 census	Asian
Asian or Asian British: Indian - England and Wales ethnic category 2011 census	Asian
Asian or Asian British: Indian - Northern Ireland ethnic category 2011 census	Asian
Asian or Asian British: Pakistani - England and Wales ethnic category 2011 census	Asian
Asian or Asian British: Pakistani - Northern Ireland ethnic category 2011 census	Asian
Asian or Asian British: any other Asian background - England and Wales ethnic category 2011 census	Asian
Asian or Asian British: any other Asian background - Northern Ireland ethnic category 2011 census	Asian
Asian or Asian Scottish or Asian British: Bangladeshi, Bangladeshi Scottish or Bangladeshi British - Scotland ethnic category 2011 census	Asian
Asian or Asian Scottish or Asian British: Chinese - Scotland ethnic category 2011 census	Asian
Asian or Asian Scottish or Asian British: Indian, Indian Scottish or Indian British - Scotland ethnic category 2011 census	Asian
Asian or Asian Scottish or Asian British: Pakistani, Pakistani Scottish or Pakistani British - Scotland ethnic category 2011 census	Asian
Asian or Asian Scottish or Asian British: any other Asian group - Scotland ethnic category 2011 census	Asian
Baltic States (Estonian or Latvian or Lithuanian) - ethnic category 2001 census	White
Bangladeshi	Asian
Bangladeshi or British Bangladeshi - ethnic category 2001 census	Asian
Black	Black
Black - ethnic group	Black
Black - other African country	Black
Black - other Asian	Black
Black - other, mixed	Mixed
Black African	Black
Black African and White	Black
Black Arab	Black
Black Black - other	Black
Black British	Black
Black British - ethnic category 2001 census	Black
Black Caribbean	Black
Black Caribbean and White	Black
Black Caribbean/W.I./Guyana	Black
Black Caribbean/West India/Guyana	Black

© 2021 Abel KM et al. *JAMA Network Open.*

Black East African Asian	Black
Black East African Asian/Indo-Caribbean	Black
Black Guyana	Black
Black Indian sub-continent	Black
Black Indo-Caribbean	Black
Black Iranian	Black
Black N African/Arab/Iranian	Black
Black North African	Black
Black West Indian	Black
Black and Asian - ethnic category 2001 census	Black
Black and Chinese - ethnic category 2001 census	Black
Black and White - ethnic category 2001 census	Black
Black or African or Caribbean or Black British: African - England and Wales ethnic category 2011 census	Black
Black or African or Caribbean or Black British: African - Northern Ireland ethnic category 2011 census	Black
Black or African or Caribbean or Black British: Caribbean - England and Wales ethnic category 2011 census	Black
Black or African or Caribbean or Black British: Caribbean - Northern Ireland ethnic category 2011 census	Black
Black or African or Caribbean or Black British: other Black or African or Caribbean background - England and Wales ethnic category 2011 census	Black
Black or African or Caribbean or Black British: other Black or African or Caribbean background - Northern Ireland ethnic category 2011 census	Black
Black, other, non-mixed origin	Black
Born in Czech Republic	White
Bosnian - ethnic category 2001 census	White
Brit. ethnic minor. spec.(NMO)	Other
Brit. ethnic minor. unsp (NMO)	Other
British Asian - ethnic category 2001 census	White
British ethnic minority specified (NMO)	Other
British ethnic minority unspecified (NMO)	Other
British or mixed British - ethnic category 2001 census	White
Bulgarian	White
Bulgarian language interpreter needed	White
Cantonese Chinese dialect	Asian
Caribbean - ethnic category 2001 census	Black
Caribbean Asian - ethnic category 2001 census	Black
Caribbean I./W.I./Guyana (NMO)	Black
Caribbean Island (NMO)	Black
Caribbean or Black: Black, Black Scottish or Black British - Scotland ethnic category 2011 census	Black
Caribbean or Black: Caribbean, Caribbean Scottish or Caribbean British - Scotland ethnic category 2011 census	Black
Caribbean or Black: any other Black or Caribbean group - Scotland ethnic category 2011 census	Black
Caucasian	White
Caucasian race	White
Caucasoid race	White
Chinese	Asian
Chinese - ethnic category 2001 census	Asian
Chinese and White - ethnic category 2001 census	Mixed
Commonwealth of (Russian) Independent States - ethnic category 2001 census	White
Cook Island Maori	Other
Cornish - ethnic category 2001 census	White
Croatian - ethnic category 2001 census	White

© 2021 Abel KM et al. *JAMA Network Open.*

Cypriot (part not stated) - ethnic category 2001 census	White
Czech	White
E Afric Asian/Indo-Carib (NMO)	Black
East African Asian (NMO)	Black
East African Asian - ethnic category 2001 census	Black
English - ethnic category 2001 census	White
Ethnic category - 2001 census	
Ethnic category - 2011 census Northern Ireland	White
Ethnic category not stated - 2001 census	
Ethnic group not recorded	
Ethnic groups (1991 census) (United Kingdom)	White
Fijian	Other
Filipino - ethnic category 2001 census	Asian
Greek (NMO)	White
Greek - ethnic category 2001 census	White
Greek Cypriot - ethnic category 2001 census	White
Guyana (NMO)	Black
Gypsies	Other
Gypsy	Other
Gypsy/Romany - ethnic category 2001 census	Other
Hungarian Roma	Other
Indian	Asian
Indian or British Indian - ethnic category 2001 census	Asian
Indian origin	Asian
Indian sub-continent (NMO)	Asian
Indo-Caribbean (NMO)	Asian
Interpreter needed - Czech	White
Interpreter needed - Vietnamese	Asian
Iranian (NMO)	Other
Iranian - ethnic category 2001 census	Other
Irish (NMO)	White
Irish - ethnic category 2001 census	White
Irish Traveller - Northern Ireland ethnic category 2011 census	Other
Irish Traveller - ethnic category 2001 census	Other
Irish traveller	Other
Israeli - ethnic category 2001 census	White
Italian - ethnic category 2001 census	White
Japanese	Asian
Japanese - ethnic category 2001 census	Asian
Kashmiri - ethnic category 2001 census	Asian
Koreans	Asian
Kosovan - ethnic category 2001 census	White
Kurdish - ethnic category 2001 census	White
Latin American - ethnic category 2001 census	Other
Main spoken language Czech	White
Main spoken language Vietnamese	Asian
Malaysian - ethnic category 2001 census	Asian
Middle Eastern (excluding Israeli, Iranian and Arab) - ethnic category 2001 census	Other
Mixed Asian - ethnic category 2001 census	Asian
Mixed Black - ethnic category 2001 census	Mixed
Mixed Irish and other White - ethnic category 2001 census	Mixed
Mixed ethnic census group	Mixed

© 2021 Abel KM et al. *JAMA Network Open.*

Mixed multiple ethnic groups: White and Asian - England and Wales ethnic category 2011 census	Mixed
Mixed multiple ethnic groups: White and Asian - Northern Ireland ethnic category 2011 census	Mixed
Mixed multiple ethnic groups: White and Black African - England and Wales ethnic category 2011 census	Mixed
Mixed multiple ethnic groups: White and Black African - Northern Ireland ethnic category 2011 census	Mixed
Mixed multiple ethnic groups: White and Black Caribbean - England and Wales ethnic category 2011 census	Mixed
Mixed multiple ethnic groups: White and Black Caribbean - Northern Ireland ethnic category 2011 census	Mixed
Mixed multiple ethnic groups: any other Mixed or multiple ethnic background - England and Wales ethnic category 2011 census	Mixed
Mixed multiple ethnic groups: any other Mixed or multiple ethnic background - Northern Ireland ethnic category 2011 census	Mixed
Mixed or multiple ethnic groups: any Mixed or multiple ethnic group - Scotland ethnic category 2011 census	Mixed
Mixed racial group	Mixed
Moroccan - ethnic category 2001 census	Other
Multi-ethnic islands: Mauritian or Seychellois or Maldivian or St Helena - ethnic category 2001 census	Mixed
N African Arab/Iranian (NMO)	Other
Nepali	Asian
New Zealand European	White
New Zealand Maori	Other
New Zealand ethnic group NOS	Other
New Zealand ethnic groups	Other
Nigerian - ethnic category 2001 census	Black
Niuean	Black
North African - ethnic category 2001 census	Other
North African Arab (NMO)	Other
Northern Irish - ethnic category 2001 census	White
Oriental	Asian
Other - ethnic category 2001 census	Other
Other African countries (NMO)	Black
Other Asian (NMO)	Asian
Other Asian background - ethnic category 2001 census	Asian
Other Asian ethnic group	Asian
Other Asian or Asian unspecified - ethnic category 2001 census	Asian
Other Black - Black/Asian orig	Black
Other Black - Black/White orig	Black
Other Black background - ethnic category 2001 census	Black
Other Black or Black unspecified - ethnic category 2001 census	Black
Other European (NMO)	White
Other European in New Zealand	White
Other Mixed background - ethnic category 2001 census	Mixed
Other Mixed or Mixed unspecified - ethnic category 2001 census	Mixed
Other New Zealand ethnic group	Other
Other Pacific ethnic group	Other
Other White European or European unspecified or Mixed European - ethnic category 2001 census	White
Other White background - ethnic category 2001 census	White
Other White or White unspecified - ethnic category 2001 census	White
Other black ethnic group	Black
Other ethnic NEC (NMO)	Other
Other ethnic group: Arab - England and Wales ethnic category 2011 census	Other

© 2021 Abel KM et al. *JAMA Network Open.*

Other ethnic group: Arab - Northern Ireland ethnic category 2011 census	Other
Other ethnic group: Arab, Arab Scottish or Arab British - Scotland ethnic category 2011 census	Other
Other ethnic group: any other ethnic group - England and Wales ethnic category 2011 census	Other
Other ethnic group: any other ethnic group - Northern Ireland ethnic category 2011 census	Other
Other ethnic group: any other ethnic group - Scotland ethnic category 2011 census	Other
Other ethnic non-mixed (NMO)	Other
Other ethnic, Asian/White orig	Asian
Other ethnic, Black/White orig	Black
Other ethnic, Black/White origin	Black
Other ethnic, mixed origin	Mixed
Other ethnic, mixed white orig	Mixed
Other ethnic, mixed white origin	Mixed
Other ethnic, other mixed orig	Mixed
Other ethnic, other mixed origin	Mixed
Other mixed White - ethnic category 2001 census	Mixed
Other republics which made up the former Yugoslavia - ethnic category 2001 census	Other
Other white British ethnic group	White
Pakeha	Other
Pakistani	Asian
Pakistani or British Pakistani - ethnic category 2001 census	Asian
Patient declined to provide information about ethnic group	
Patient ethnicity unknown	
Polish - ethnic category 2001 census	White
Polish Roma	Other
Portuguese	White
Punjabi - ethnic category 2001 census	Asian
RACE: Bangladeshi	Asian
RACE: Chinese	Asian
RACE: Pakistani	Asian
Race: West indian	Black
Race: White	White
Refusal by patient to provide information about ethnic group	
Romanian	White
Romanian Roma	Other
Samoan	Other
Scottish - ethnic category 2001 census	White
Serbian - ethnic category 2001 census	White
Sinhalese - ethnic category 2001 census	Asian
Slovak	White
Slovak Roma	Other
Somali - ethnic category 2001 census	Black
South East Asian	Asian
South and Central American - ethnic category 2001 census	Other
Sri Lankan - ethnic category 2001 census	Asian
Tamil - ethnic category 2001 census	Asian
Tokelauan	Other
Tongan	Other
Traveller - ethnic category 2001 census	Other
Turkish (NMO)	White
Turkish - ethnic category 2001 census	White
Turkish Cypriot (NMO)	White
Turkish Cypriot - ethnic category 2001 census	White

© 2021 Abel KM et al. *JAMA Network Open.*

Turkish/Turkish Cypriot (NMO)	White
Ulster Scots - ethnic category 2001 census	White
Unknown racial group	
Vietnamese	Asian
Vietnamese - ethnic category 2001 census	Asian
Vietnamese language	Asian
Welsh - ethnic category 2001 census	White
West Indian (NMO)	Black
West Indian origin	Black
White	White
White - Northern Ireland ethnic category 2011 census	White
White - ethnic group	White
White British	White
White British - ethnic category 2001 census	White
White Irish	White
White Irish - ethnic category 2001 census	White
White Scottish	White
White and Asian - ethnic category 2001 census	Mixed
White and Black African - ethnic category 2001 census	Mixed
White and Black Caribbean - ethnic category 2001 census	Mixed
White: English or Welsh or Scottish or Northern Irish or British - England and Wales ethnic category 2011 census	White
White: Gypsy or Irish Traveller - England and Wales ethnic category 2011 census	Other
White: Gypsy or Irish Traveller - Scotland ethnic category 2011 census	Other
White: Irish - England and Wales ethnic category 2011 census	White
White: Irish - Scotland ethnic category 2011 census	White
White: Polish - Scotland ethnic category 2011 census	White
White: Scottish - Scotland ethnic category 2011 census	White
White: any other White background - England and Wales ethnic category 2011 census	White
White: any other White ethnic group - Scotland ethnic category 2011 census	White
White: other British - Scotland ethnic category 2011 census	White
Yemeni	Other
Bangladeshi	Asian
Black	Black
Black	Black
Black Caribbean	Black
Bulgarian	White
Bulgarian	White
Caucasian	White
Chinese	Asian
Chinese	Asian
Czech	White
Fijian	Other
Indian	Asian
Indian	Asian
Japanese	Asian
Pakistani	Asian
Portuguese	White
Portuguese	White
Romanian	White
Samoan	Other
Slovak	White
Tongan	Other

© 2021 Abel KM et al. *JAMA Network Open.*

Vietnamese	Asian
White	White
Yemeni	Other

© 2021 Abel KM et al. *JAMA Network Open.*

eFigure 1. Flowchart Showing Selection in to Matched Cohorts

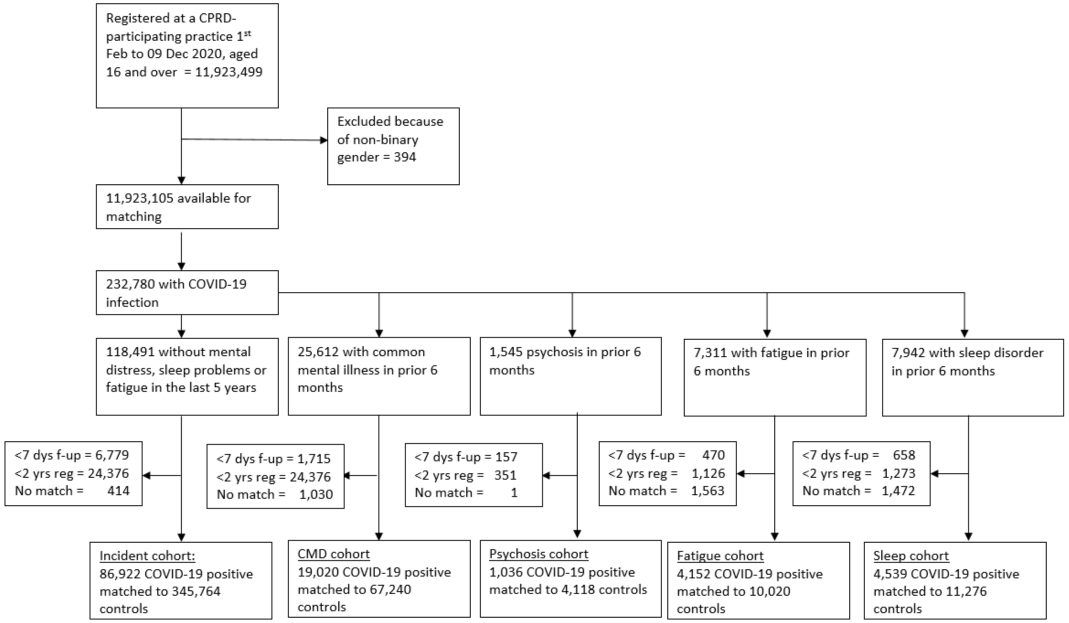

© 2021 Abel KM et al. *JAMA Network Open.*

eFigure 2. Cumulative Hazard and Scaled Schoenfeld Residual Plots

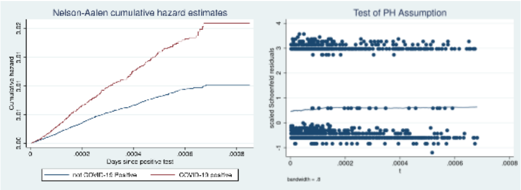

Incident cohort: any psychiatric morbidity

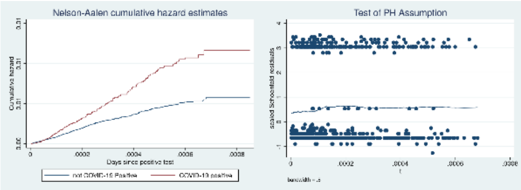

Incident cohort: depression

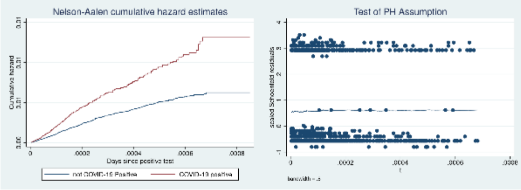

Incident cohort: anxiety

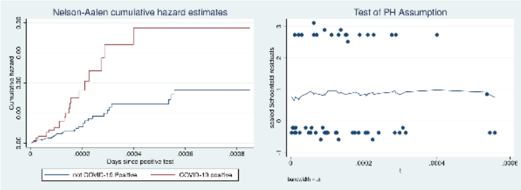

Incident cohort: psychosis

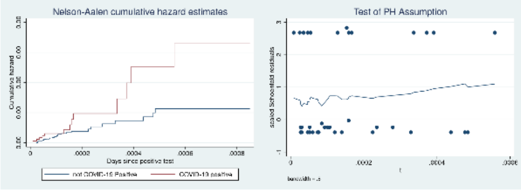

Incident cohort: self-harm

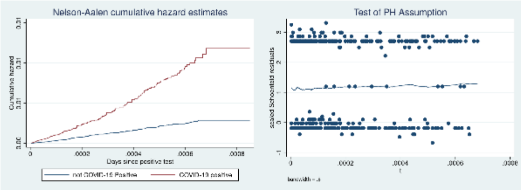

Incident cohort: sleep

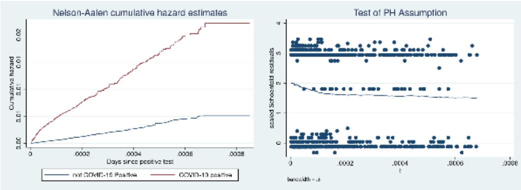

Incident cohort: fatigue

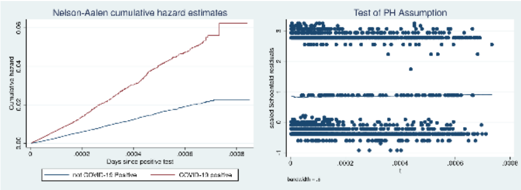

Incident cohort: any psychotropic medications

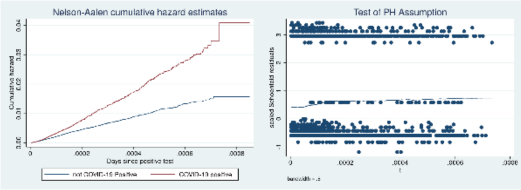

Incident cohort: antidepressants

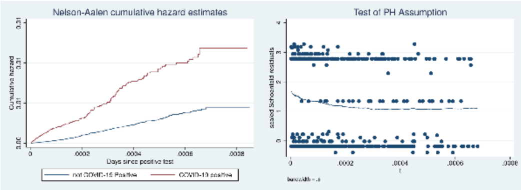

Incident cohort: benzodiazepines

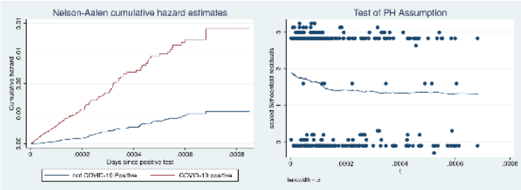

Incident cohort: Nonbenzodiazepine hypnotics

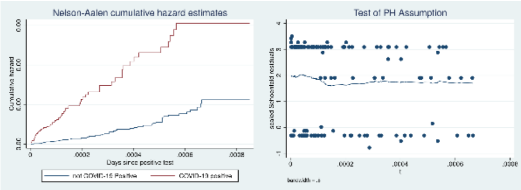

Incident cohort: antipsychotics

© 2021 Abel KM et al. *JAMA Network Open.*

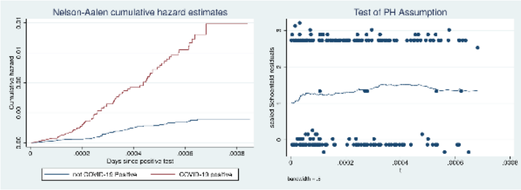

Incident cohort: mood stabilisers

© 2021 Abel KM et al. *JAMA Network Open.*

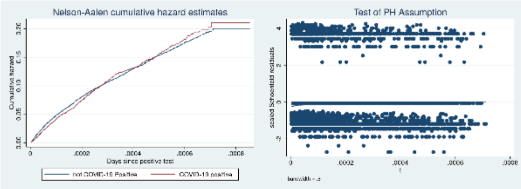

Common mental disorder cohort: depression

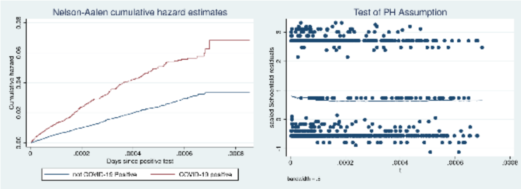

Common mental disorder cohort: benzodiazepines

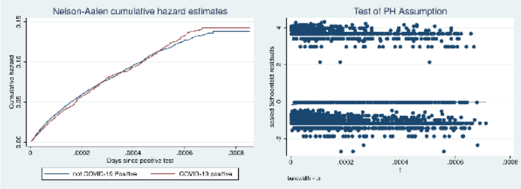

Common mental disorder cohort: anxiety

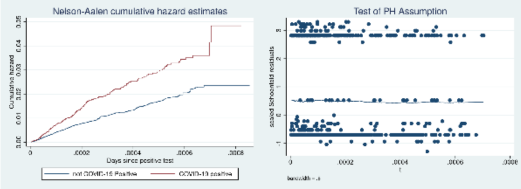

Common mental disorder cohort: Nonbenzodiazepine hypnotics

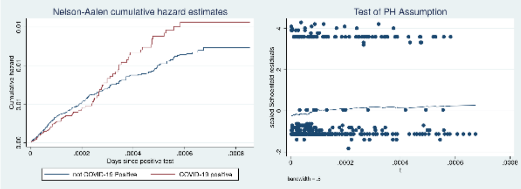

Common mental disorder cohort: psychosis

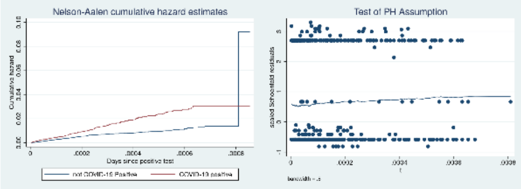

Common mental disorder cohort: antipsychotics

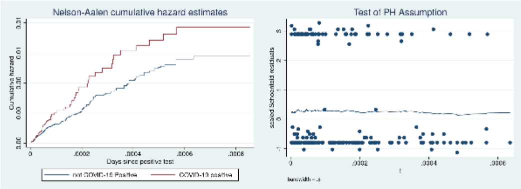

Common mental disorder cohort: self-harm

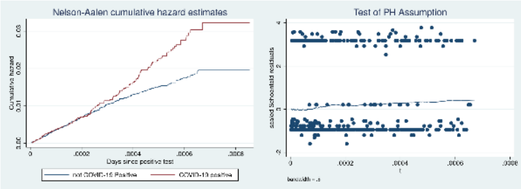

Common mental disorder cohort: mood stabilisers

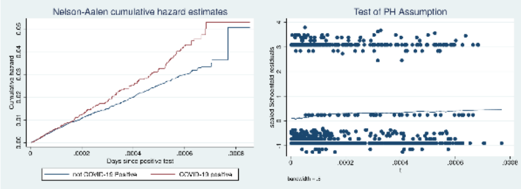

Common mental disorder cohort: Sleep problems

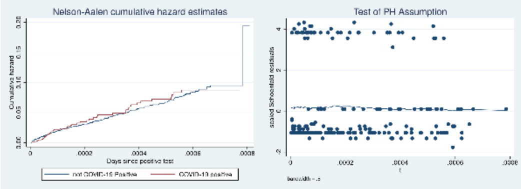

Psychosis cohort: depression

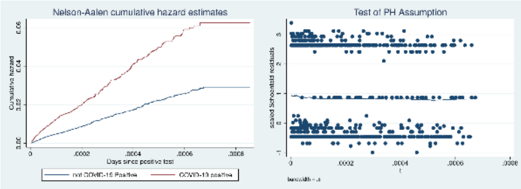

Common mental disorder cohort: fatigue

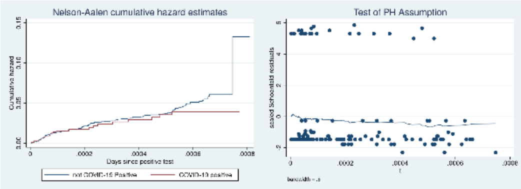

Psychosis cohort: anxiety

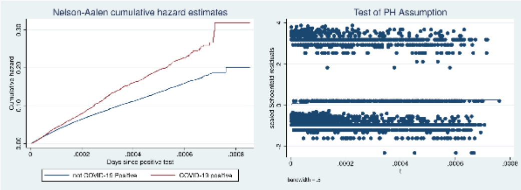

Common mental disorder cohort: antidepressants

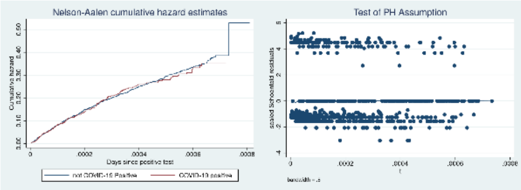

Psychosis cohort: psychosis

© 2021 Abel KM et al. *JAMA Network Open.*

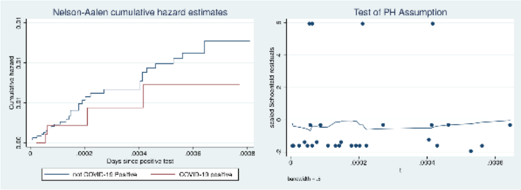

Psychosis cohort: self-harm

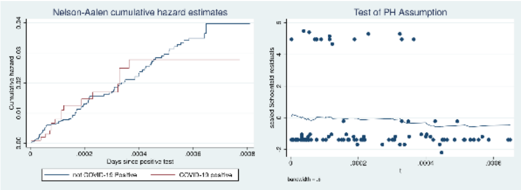

Psychosis cohort: sleep disorder

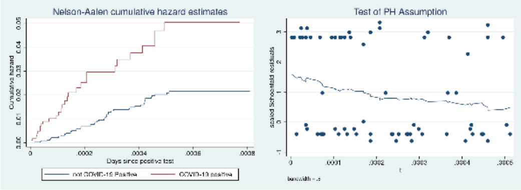

Psychosis cohort: fatigue

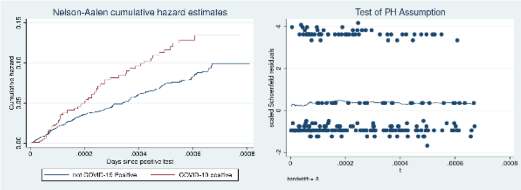

Psychosis cohort: antidepressants

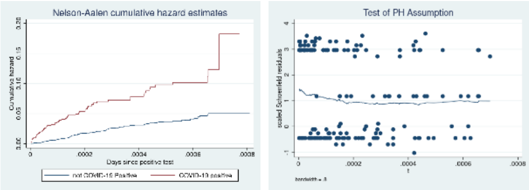

Psychosis cohort: benzodiazepines

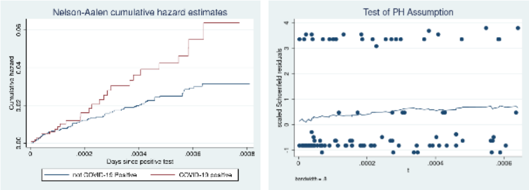

Psychosis cohort: Nonbenzodiazepine hypnotics

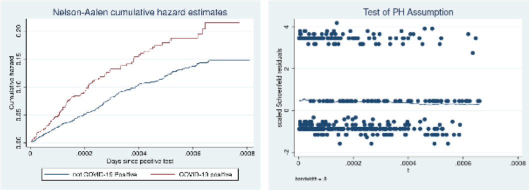

Psychosis cohort: antipsychotics

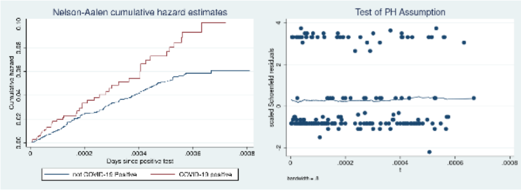

Psychosis cohort: mood stabilisers

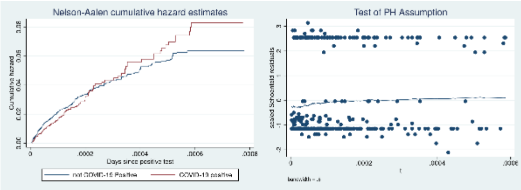

Fatigue cohort: depression

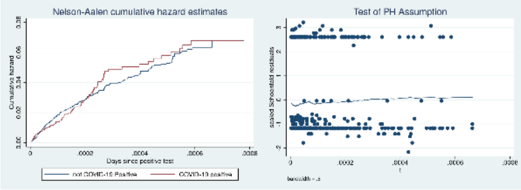

Fatigue cohort: anxiety

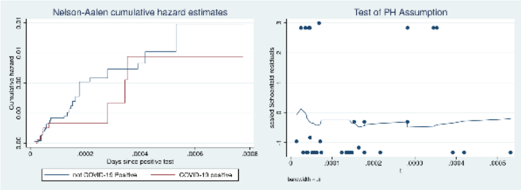

Fatigue cohort: psychosis

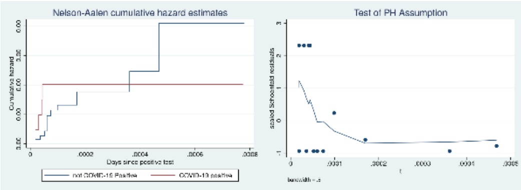

Fatigue cohort: self-harm

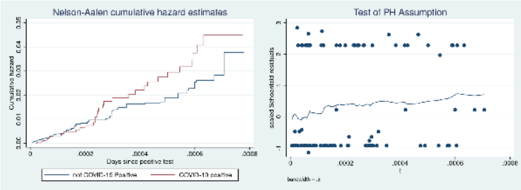

Fatigue cohort: sleep problems

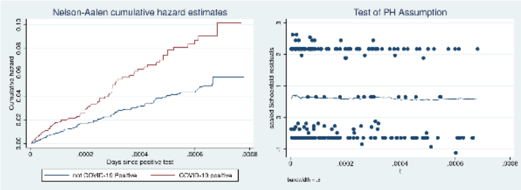

Fatigue cohort: fatigue

© 2021 Abel KM et al. *JAMA Network Open.*

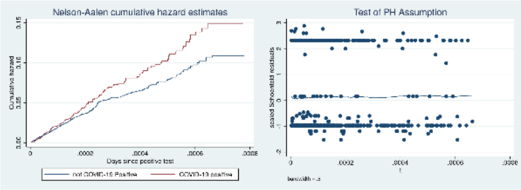

Fatigue cohort: antidepressants

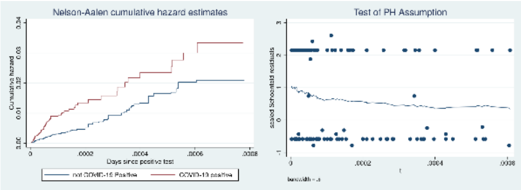

Fatigue cohort: benzodiazepines

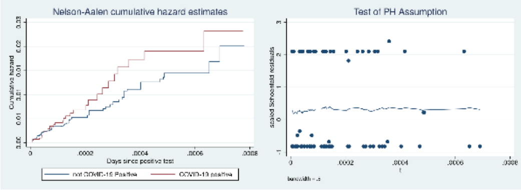

Fatigue cohort: Nonbenzodiazepine hypnotics

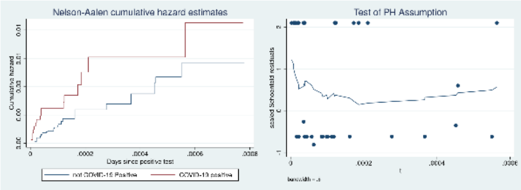

Fatigue cohort: antipsychotics

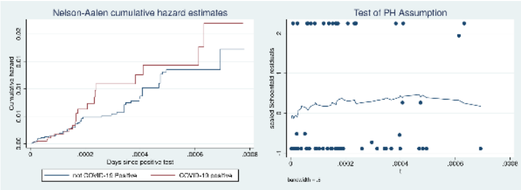

Fatigue cohort: mood stabilisers

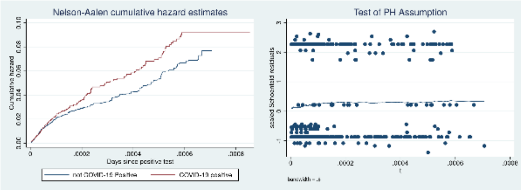

Sleep cohort: depression

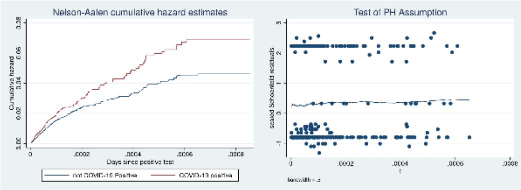

Sleep cohort: anxiety

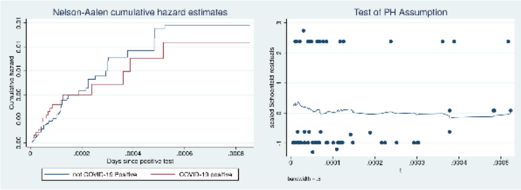

Sleep cohort: psychosis

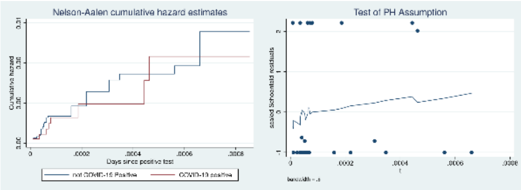

Sleep cohort: self-harm

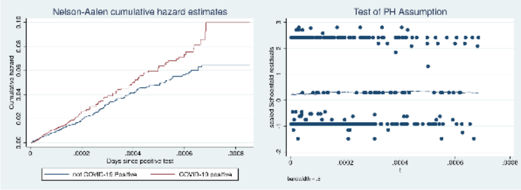

Sleep cohort: sleep problems

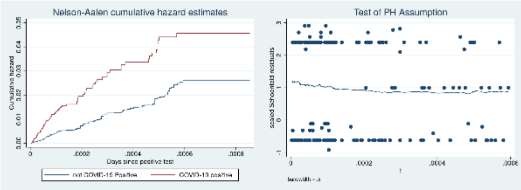

Sleep cohort: fatigue

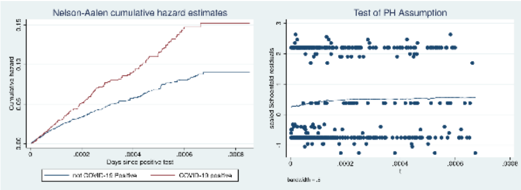

Sleep cohort: antidepressants

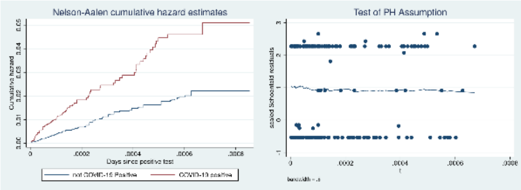

Sleep cohort: benzodiazepines

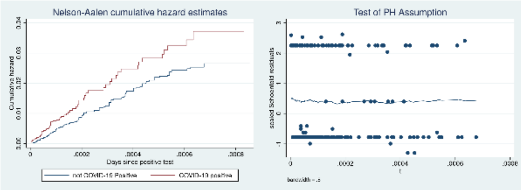

Sleep cohort: nonbenzodiazepine hypnotics

© 2021 Abel KM et al. *JAMA Network Open.*

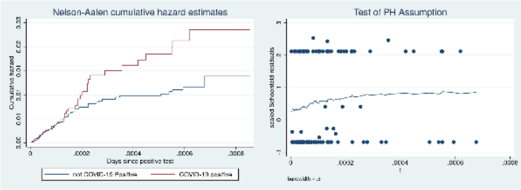

Sleep cohort: antipsychotics

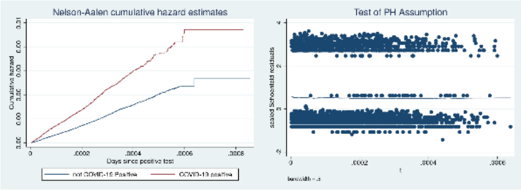

Negative test cohort: sleep problems

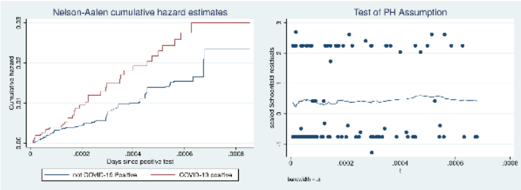

Sleep cohort: mood stabilisers

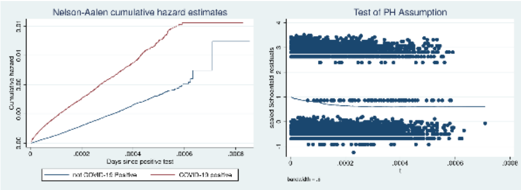

Negative test cohort: fatigue

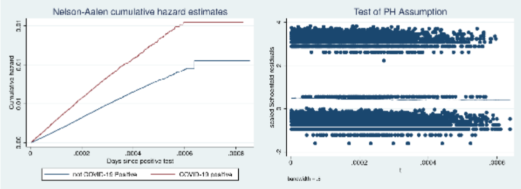

Negative test cohort: Any psychiatric morbidity

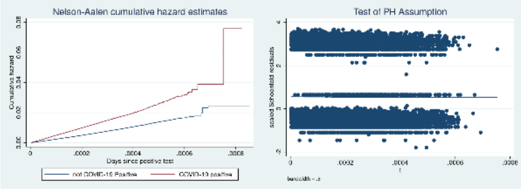

Negative test cohort: any psychotropic medication

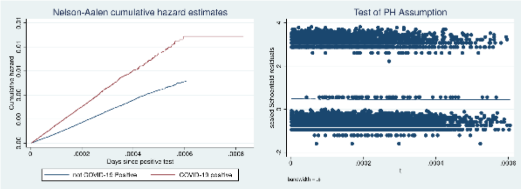

Negative test cohort: depression

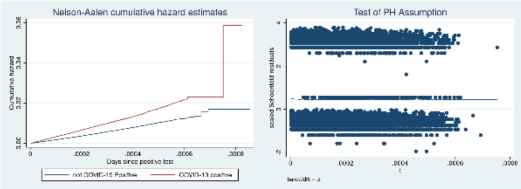

Negative test cohort: antidepressants

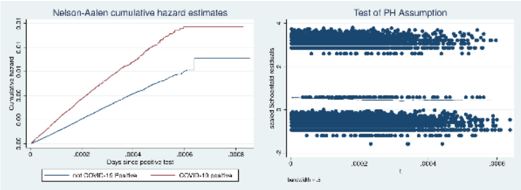

Negative test cohort: anxiety

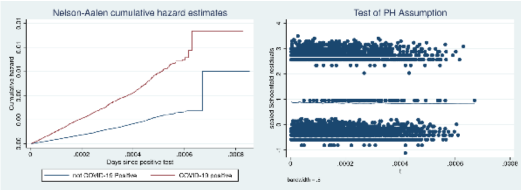

Negative test cohort: benzodiazepines

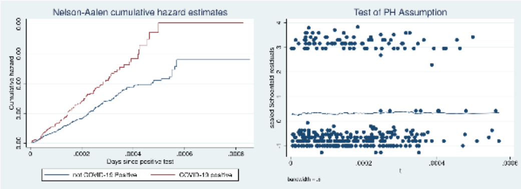

Negative test cohort: psychosis

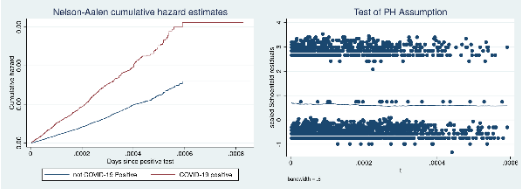

Negative test cohort: Nonbenzodiazepine hypnotics

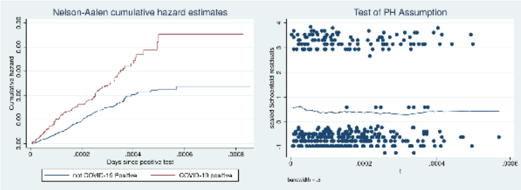

Negative test cohort: self-harm

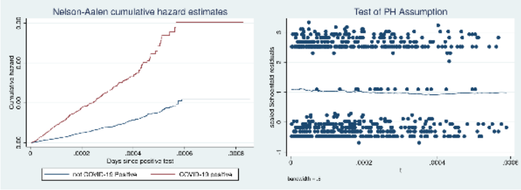

Negative test cohort: antipsychotics

© 2021 Abel KM et al. *JAMA Network Open.*

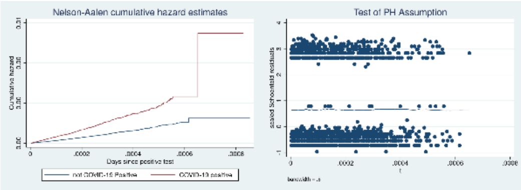

Negative test cohort: mood stabilisers

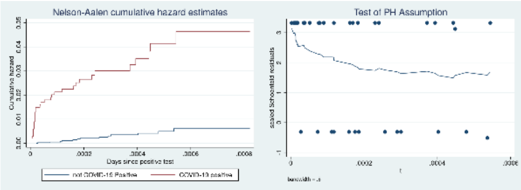

Flu cohort: fatigue

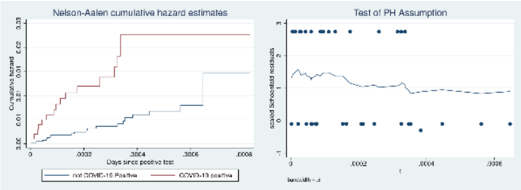

Flu cohort: any psychiatric morbidity

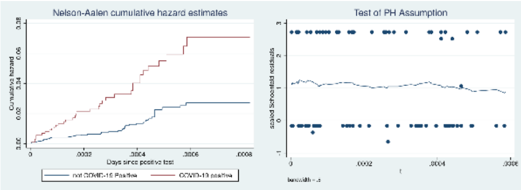

Flu cohort: any psychotropic medications

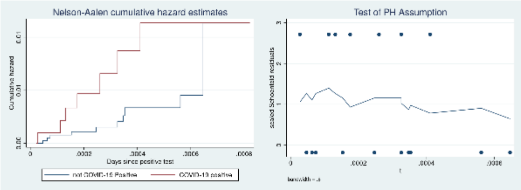

Flu cohort: depression

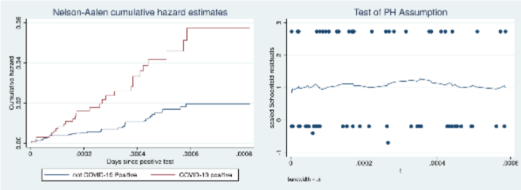

Flu cohort: antidepressants

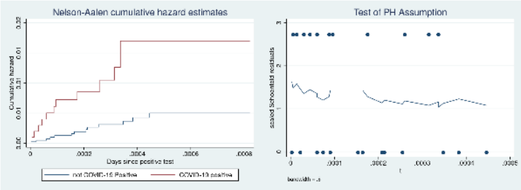

Flu cohort: anxiety

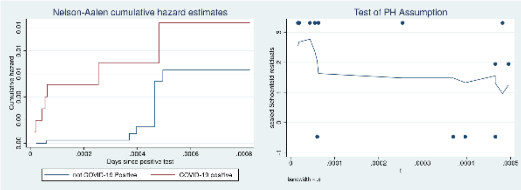

Flu cohort: benzodiazepines

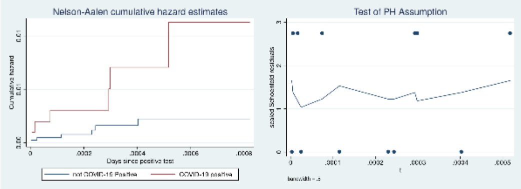

Flu cohort: sleep problems

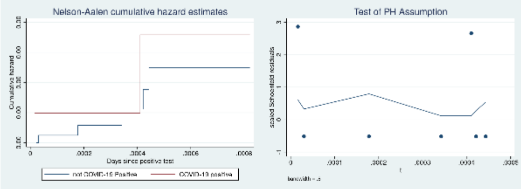

Flu cohort: Nonbenzodiazepine hypnotics

© 2021 Abel KM et al. *JAMA Network Open.*

eFigure 3. Histogram of Frequency of Positive SARS-CoV-2 Test Results by Date During 2020

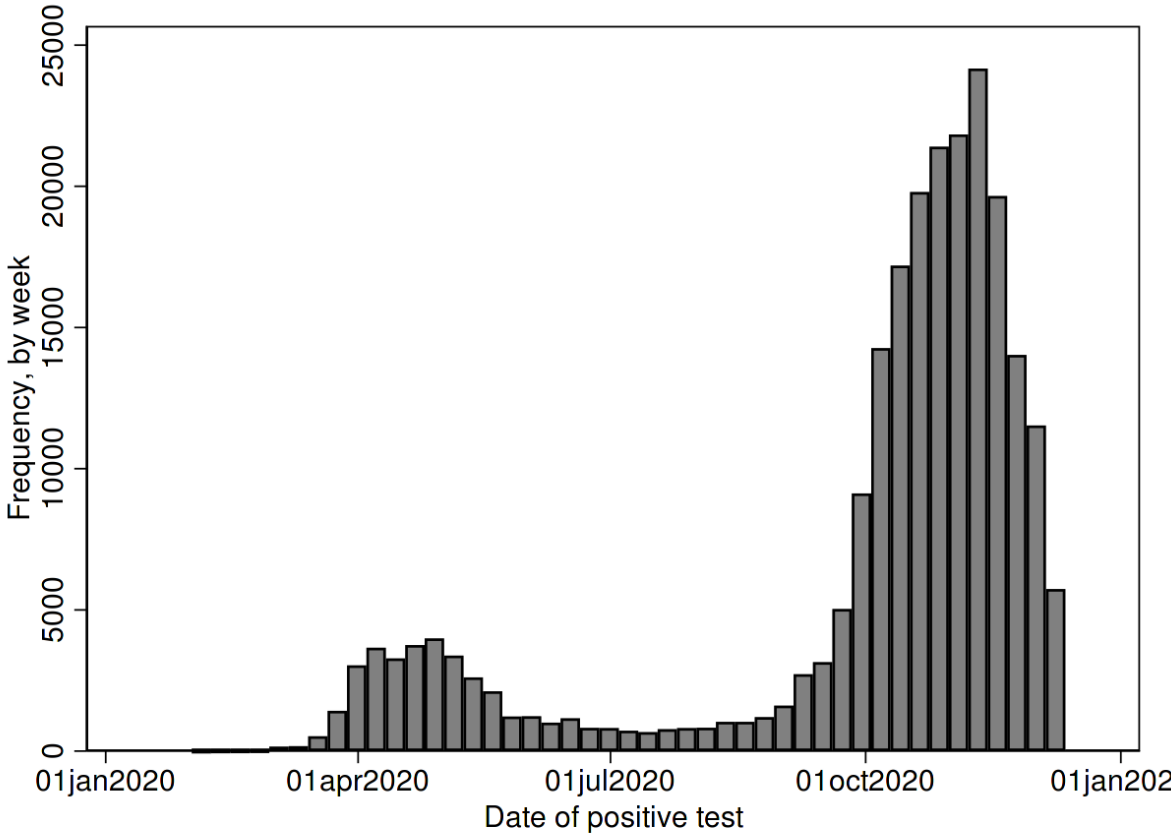

© 2021 Abel KM et al. *JAMA Network Open.*

eTable 2. Proportion of Individuals With Positive Test Results and Controls With Outcomes After 6 Months Within Each Matched Cohort

	Matched cohort									
	Incident		CMD		Psychosis		Fatigue		Sleep	
Outcome	COVID-19	Controls	COVID-19	Controls	COVID-19	Controls	COVID-19	Controls	COVID-19	Controls
Any psychological distress	1.44	0.85	20.26	20.18	30.23	31.42	9.71	7.65	10.13	9.29
Depression	0.85	0.47	14.51	14.59	6.99	6.13	6.84	5.04	6.55	6.19
Anxiety	0.91	0.53	10.70	11.21	3.51	3.78	5.79	3.86	5.64	5.68
Psychosis	0.07	0.03	1.37	1.04	24.50	26.07	0.70	0.78	0.57	0.59
Self-harm	0.05	0.02	0.68	0.55	0.72	0.95	0.43	0.24	0.10	0.24
Fatigue	1.37	0.36	4.87	2.17	4.93	1.93	4.19	1.71	7.18	4.16
Sleep	0.72	0.21	3.18	2.43	2.74	2.97	6.05	4.63	2.71	1.71
Any psychotropic medication	3.54	1.61	83.35	83.11	94.45	94.62	42.21	34.86	31.89	29.42
Antidepressants	2.09	1.14	79.55	80.23	49.98	50.41	33.45	29.32	28.72	25.91
Benzodiazepines	0.80	0.30	8.85	6.74	20.50	14.94	6.55	3.40	3.39	2.91
Nonbenzodiazepine hypnotics	0.52	0.15	5.64	4.99	11.04	10.01	5.45	3.84	3.00	2.31
Antipsychotics	0.20	0.06	5.65	5.35	72.75	75.07	4.17	3.42	1.72	1.59
Mood stabilisers	0.41	0.12	8.29	7.87	40.45	36.79	8.00	6.31	4.77	4.36

© 2021 Abel KM et al. *JAMA Network Open.*

eTable 3. Psychiatric Morbidity, Sleep Problems, Fatigue and Uniquely Prescribed Psychotropic Medications for Those With Preexisting Common Mental Illness, Psychosis, Fatigue, or Sleep Problems Matched on Year of Birth, Sex, and General Practice

	Common mental illness			Psychosis			Fatigue			Sleep		
	Rate			Rate			Rate			Rate		
Outcome	COVID-19	Controls	aHR [95 % CI]	COVID-19	Controls	aHR [95 % CI]	COVID-19	Controls	aHR [95 % CI]	COVID-19	Controls	aHR [95 % CI]
Depression	352.5	379.8	0.94 [0.89, 0.99]	155.3	138.1	1.20 [0.85, 1.69]	149.0	167.2	0.98 [0.77, 1.25]	172.2	132.9	1.35 [1.09, 1.67]
Anxiety	263.1	280.8	1.00 [0.94, 1.07]	77.9	94.1	0.99 [0.59, 1.68]	131.0	145.0	0.86 [0.65, 1.14]	138.0	95.0	1.42 [1.09, 1.86]
Psychosis	24.9	24.6	1.73 [1.08, 2.76]	623.4	639.6	1.03 [0.87, 1.21]	10.1	13.3	1.54 [0.69, 3.44]	19.3	17.3	0.69 [0.26, 1.78]
Self-harm	15.8	11.6	1.31 [0.93, 1.85]	13.4	20.3	0.87 [0.24, 3.11]	5.1	4.6	3.68 [0.45, 30.43]	8.1	6.9	1.04 [0.51, 2.13]
Sleep disorders	65.9	51.8	1.24 [1.07, 1.43]	54.2	60.8	0.77 [0.39, 1.49]	48.4	37.6	1.23 [0.75, 2.03]	123.8	94.8	1.32 [1.06, 1.64]
Fatigue	105.6	44.5	2.24 [1.99, 2.53]	95.8	32.4	3.84 [2.02, 7.29]	153.4	84.2	2.17 [1.74, 2.71]	92.0	36.7	3.22 [2.35, 4.40]
Antidepressants	398.8	323.8	1.20 [1.13, 1.26]	227.5	164.7	1.64 [1.23, 2.20]	222.7	186.5	1.17 [0.97, 1.41]	243.7	149.8	1.44 [1.21, 1.70]
Benzodiazepines	108.9	52.9	1.91 [1.68, 2.17]	225.5	79.4	2.96 [2.03, 4.31]	67.5	33.5	1.68 [0.95, 2.97]	87.5	32.8	2.68 [1.86, 3.84]
Non-benzodiazepine hypnotics	64.0	39.0	1.87 [1.59, 2.19]	88.6	56.9	2.44 [1.43, 4.15]	38.2	27.2	1.14 [0.74, 1.77]	63.7	39.4	1.56 [1.10, 2.21]
Antipsychotics	46.4	23.0	2.29 [1.77, 2.95]	381.3	252.8	1.74 [1.39, 2.17]	20.3	10.8	1.75 [0.81, 3.80]	39.8	20.8	1.82 [0.56, 5.86]
Mood stabilisers	41.4	34.5	1.19 [0.98, 1.45]	158.3	108.7	1.03 [0.70, 1.53]	26.7	22.6	1.02 [0.73, 1.42]	45.0	27.4	1.48 [0.91, 2.40]

aHR = adjusted hazard ratios

Rate per 1,000 person years

Note: all medication outcomes refer to prescriptions not previously prescribed 6 months prior to index date

© 2021 Abel KM et al. *JAMA Network Open.*

eTable 4. Comparison of Adjusted Hazard Ratios From Matched Positive SARS-CoV-2 Test Results, Negative SARS-CoV-2 Test Results, and Influenza Cohorts

	**Matched cohort**		
**Outcome**	**Positive**	**Negative test**	**Flu**
Any psychological distress	1.83 [1.66, 2.02]	1.71 [1.65, 1.77]	2.98 [1.55, 5.75]
Depression	1.74 [1.51, 2.00]	1.71 [1.63, 1.79]	3.63 [1.31, 10.06]
Anxiety	1.93 [1.71, 2.18]	1.76 [1.69, 1.84]	3.65 [1.71, 7.81]
Psychosis	1.84 [0.93, 3.64]	1.40 [1.11, 1.77]	*
Self-harm	2.21 [1.11, 4.39]	1.76 [1.43, 2.16]	*
Sleep disorders	3.16 [2.64, 3.78]	1.79 [1.67, 1.91]	4.72 [1.71, 13.04]
Fatigue	5.98 [5.33, 6.71]	2.34 [2.23, 2.46]	22.72 [7.55, 68.32]
Any psychotropic medication	2.24 [2.09, 2.40]	1.81 [1.76, 1.85]	2.77 [1.82, 4.21]
Antidepressants	1.72 [1.57, 1.88]	1.69 [1.64, 1.75]	2.80 [1.82, 4.32]
Benzodiazepines	3.50 [2.95, 4.15]	2.19 [2.06, 2.34]	20.67 [2.45, 174.65]
Nonbenzodiazepine hypnotics	4.90 [4.00, 5.99]	1.98 [1.82, 2.15]	1.67 [0.32, 8.71]
Antipsychotics	7.61 [5.00, 11.60]	2.29 [1.96, 2.69]	*
Mood stabilisers	3.55 [2.74, 4.61]	2.16 [1.96, 2.39]	*

*Insufficient events (≤5) to fit model

© 2021 Abel KM et al. *JAMA Network Open.*

eTable 5. Description of Eligible Cohort According to SARS-CoV-2 Test and Influenza Status Over Follow-up

**Characteristic**	**COVID positive only, N=159,155**	**COVID negative only, N= 1,464,693**	**COVID positive and negative, N= 67,366**	**Flu, N= 36,913**	**Not COVID positive negative or flu, N=10,194,978**
Sex					
Female	84,367 (53.0)	854,045 (58.3)	42,614 (63.3)	22,633 (61.3)	5,007,361 (49.1)
Male	74,788 (47.0)	610,648 (41.7)	24,752 (36.7)	14,280 (38.7)	5,187,617 (50.9)
Median age [IQR]	42 [27, 57]	40 [29, 55]	42 [28, 57]	48 [35, 61]	45 [31, 62]
Ethnicity					
White	103,992 (65.3)	1,020,184 (69.7)	45,000 (66.8)	24,183 (65.5)	6,505,441 (63.8)
Asian	17,630 (11.1)	105,892 (7.2)	7,140 (10.6)	4,792 (13.0)	887,280 (8.7)
Black	5,208 (3.3)	44,017 (3.0)	2,052 (3.1)	1,994 (5.4)	454,195 (4.5)
Mixed	2,323 (1.5)	20,710 (1.4)	919 (1.4)	637 (1.7)	163,957 (1.6)
Other	1,889 (1.2)	14,181 (1.0)	667 (1.0)	558 (1.5)	161,045 (1.6)
Missing	28,113 (17.7)	259,709 (17.7)	11,588 (17.2)	4,749 (12.9)	2,023,060 (19.8)
Median BMI [IQR]	26.4 [23.0-30.7]	26.0 [22.6-30.1]	26.2 [22.7-30.8]	27.0 [23.4-31.6]	25.8 [22.6-29.7]
Psychiatric illness in the last 5 years					
Depression	23,924 (15.0)	294,753 (20.1)	13,460 (20.0)	9,566 (25.9)	1,298,455 (12.7)
Anxiety disorders	19,502 (12.3)	238,155 (16.3)	10,573 (15.7)	7,178 (19.5)	1,027,132 (10.1)
Psychosis	1,542 (1.0)	18,989 (1.3)	1,088 (1.6)	708 (1.9)	107,745 (1.1)
Eating disorder	852 (0.5)	8,963 (0.6)	461 (0.7)	285 (0.8)	37,801 (0.4)
Personality disorder	418 (0.3)	7,387 (0.5)	270 (0.4)	358 (1.0)	34,824 (0.3)
Self-harm	1,825 (1.2)	25,893 (1.8)	1,051 (1.6)	896 (2.4)	111,136 (1.1)
Fatigue	11,581 (7.3)	119,886 (8.2)	6,095 (9.1)	4,662 (12.6)	541,824 (5.3)
Sleep disorder	10,737 (6.8)	105,440 (7.2)	5,338 (7.9)	4,339 (11.8)	560,433 (5.5)
Medication in the last 5 years					
Antidepressants	40,539 (25.5)	458,703 (31.3)	22,414 (33.3)	15,384 (41.7)	2,209,707 (21.7)
Benzodiazepines	12,286 (7.7)	144,620 (9.9)	7,395 (11.0)	5,669 (15.4)	677,395 (6.6)
Nonbenzodiazepine hypnotics	7,598 (4.8)	91,944 (6.3)	4,426 (6.6)	3,773 (10.2)	447,420 (4.4)
Antipsychotics	2,765 (1.7)	35,933 (2.5)	2,256 (3.4)	1,352 (3.7)	172,385 (1.7)
Mood stabilisers	9,518 (6.0)	93,785 (6.4)	5,106 (7.6)	4,152 (11.3)	489,268 (4.8)

Note that if reported flu over follow-up then patient was in flu category, regardless of other exposures.

© 2021 Abel KM et al. *JAMA Network Open.*

eTable 6. Estimates for Individuals With Positive SARS-CoV-2 Test Results in the First or Second Wave

		Exposed		Unexposed			
**Outcome**	**Period of infection**	**Pyears**	**Rate**	**Pyears**	**Rate**	**aHR**	**p-value**
Any psychological distress	1st wave	6.3	33.9	26.4	14.6	2.48 [2.13, 2.87]	<0.0001
	2nd wave	8.4	27.5	33.7	19.6	1.49 [1.30, 1.70]	
Sleep disorders	1st wave	6.4	18.2	26.5	4.2	4.21 [3.26, 5.43]	0.002
	2nd wave	8.4	8.8	33.7	3.7	2.39 [1.85, 3.08]	
Fatigue	1st wave	6.3	44.9	26.5	7.3	6.42 [5.42, 7.61]	0.253
	2nd wave	8.4	35.1	33.7	6.5	5.63 [4.83, 6.57]	
Any psychotropic medication	1st wave	6.2	106.2	26.3	32.0	3.14 [2.86, 3.45]	<0.0001
	2nd wave	8.4	46.9	33.7	29.9	1.57 [1.42, 1.74]	

p-value testing for equivalence of adjusted hazard ratios between first and second wave

© 2021 Abel KM et al. *JAMA Network Open.*

eTable 7. Comparison of Estimates From Main Adjusted Analysis of the Incident Cohort With That Calculated Controlling for a Propensity Score

Outcome	Main analysis	Propensity score control
Any psychological distress	1.74 [1.51, 2.00]	1.77 [1.61, 1.95]
Sleep disorders	3.16 [2.64, 3.78]	2.99 [2.53, 3.54]
Fatigue	5.98 [5.33, 6.71]	5.80 [5.19. 6.48]
Any psychotropic medication	2.24 [2.09, 2.40]	2.18 [2.04, 2.33]

© 2021 Abel KM et al. *JAMA Network Open.*

eTable 8. Estimates From the Main Analysis for Depression, Anxiety, and Psychosis and After Including Only Diagnosis Codes in the Outcome Definition

Outcome	Outcome definition	Rate COVID-19	Rate unexposed	**HR**	**aHR**
Depression	All	15.52	9.49	1.71 [1.50, 1.95]	1.74 [1.51, 2.00]
	Diagnosis	11.53	6.98	1.61 [1.38, 1.88]	1.65 [1.41, 1.94]
Anxiety	All	20.12	11.15	1.85 [1.65, 2.08]	1.93 [1.71, 2.18]
	Diagnosis	8.15	4.61	1.72 [1.43, 2.06]	1.90 [1.57, 2.30]
Psychosis	All	1.41	0.60	2.34 [1.48, 3.70]	1.84 [0.93, 3.64]
	Diagnosis	0.54	0.43	1.15 [0.57, 2.32]	0.89 [0.33, 2.39]

Rate per 1,000 person years

© 2021 Abel KM et al. *JAMA Network Open.*

eTable 9. Repeating the Incident Matched Analysis for Individuals With Recent Clinical Contact, as Indicated by Recording of Routine Clinical Data

	Original			Contact within 2 years			Contact within 1 year			Contact within 6 months		
	Rate			Rate			Rate			Rate		
Outcome	COVID-19	Un-exposed	aHR [95 % CI]	COVID-19	Un-exposed	aHR [95 % CI]	COVID-19	Un-exposed	aHR [95 % CI]	COVID-19	Un-exposed	aHR [95 % CI]
Any psychiatric morbidity	30.24	17.39	1.83 [1.66, 2.02]	34.26	20.36	1.74 [1.54, 1.96]	35.15	20.25	1.81 [1.57, 2.07]	37.99	23.80	1.60 [1.37, 1.87]
Sleep disorders	12.82	3.92	3.16 [2.64, 3.78]	14.65	6.10	2.23 [1.82, 2.72]	15.67	6.32	2.43 [1.95, 3.03]	17.93	6.49	2.91 [2.23, 3.80]
Fatigue	39.29	6.88	5.98 [5.33, 6.71]	43.69	9.13	4.85 [4.24, 5.55]	44.49	8.87	5.02 [4.32, 5.85]	47.13	8.94	5.27 [4.40, 6.32]
Any psychotropic medication	72.05	30.82	2.24 [2.09, 2.40]	85.41	37.88	2.20 [2.03, 2.39]	90.19	38.25	2.24 [2.05, 2.46]	92.79	42.74	2.11 [1.90, 2.35]

© 2021 Abel KM et al. *JAMA Network Open.*
